# Supplementary material for: Assessing a bundle of peer counseling, mobile phone messages, and mama kits in promoting timely initiation of and exclusive breastfeeding in Uganda: A cluster randomized controlled study
Source: PLoS One. 2025 Jan 24;20(1):e0317200. doi: 10.1371/journal.pone.0317200 (PMC11761178; doi:10.1371/journal.pone.0317200)
Supplement: S4 File — (PDF) [file pone.0317200.s004.pdf]

THE SURVIVAL PLUSS STUDY

SURVIVAL PLUSS: Increasing capacity for mama-baby survival in post-conflict Uganda and South Sudan

Investigators:

Principal Investigator: James K Tumwine[1] (MBChB, MMED, PhD)

Co-Investigators: Grace Ndeezi[1] (MBChB, MMED, PhD), Victoria Nankabirwa[1] (MBChB, MPH, PhD), Elizabeth Bwanga[2] (BA, MA,PhD), Paul Waako[3] (MBChB, MSc, PhD), Joyce Kaducu[4] (MBChB, MMED), Jino David[5] (MBBS, MSc), Mohammed Boy Sebit[5] (MBBS, MMED, PhD), Thorkild Tylleskar[6] (MD, PhD).

Collaborators:

Fredrick Froen (MD, PhD)[7], Jagrati Jani Bolstad (MD, PhD)[7], Halvor Sommerfelt[6] (MD, PhD).

Institutions:

1. College of Health Sciences, Makerere University,
2. College of Humanities and Social Sciences, Makerere University
3. Busitema University
4. Gulu University Medical School
5. Juba University Medical School, South Sudan,
6. Center for International Health, University of Bergen, Norway,
7. Norwegian Institute of Public Health

Table of contents

Operational definitions **4**
Abstract **6**
Introduction **7**
Problem statement and justification of the study **10**
Research question **11**
Objectives **11**
Conceptual framework **12**
Methods **14**
- **Study design** 14
- **Study site** 14
- **Participants** 15
  - **Inclusion criteria and exclusion criteria** 16

Recruitment **16**
The Intervention **16**
Sample size **18**
Randomization **18**
Measurements **18**
- **Primary outcome** 18
- **Secondary outcomes** 19
- **Baseline characteristics and potential confounders** 19
- **Infant feeding practices** 19
- **Anthropometric measurements** 19
- **Morbidity and mortality** 19

Data collection and Management **20**
Quality control **21**
Statistical methods **22**
Ethical issues **23**
Dissemination of findings **24**
References **25**
Appendices **27**
- **Consent forms** 27
- Questionnaires 34

List of abbreviations

ANC	Antenatal care
BCG	Bacille Calmette-Guérin
BMS	Between subject mean square
CI	Confidence interval
DHS	Demographic and health survey
DPT-HB-Hib	Diphteria-pertussis-tetanus-hepatitis B-haemophilus influenza b
EBF	Exclusive breastfeeding
EMS	Error mean square
ENM	Early neonatal mortality
ENMR	Early neonatal mortality risk
GLM	Generalised linear model
HAZ	Height for age Z scores
HIC	High income country
HIV	Human immunodeficiency virus
IM	Infant mortality
IMR	Infant mortality rate
IQR	Inter quartile range
JMS	Judge mean square
LAZ	Length for age Z scores
LMIC	Low and middle income countries
MCA	Multiple component analysis
NMR	Neonatal mortality rate
PCA	Principle component analysis
PCR	Polymerase chain reaction
PMR	Perinatal mortality rate
PNMR	Post-neonatal mortality rate
PR	Prevalence ratio
RCT	Randomised controlled trial
RR	Risk ratio (relative risk)
SBR	Stillbirth risk
SES	Socio-economic status
TBA	Traditional birth attendant
U5MR	Under 5 mortality rate
UDHS	Uganda demographic and health survey
UNEPI	Uganda national expanded program on immunization
WAZ	Weight for age Z scores
WHO	World health organization
WHZ	Weight for height Z scores
WLZ	Weight for length Z scores

Operational definitions

1) **Types of death**

Stillbirth: Death prior to complete expulsion from the mother of a fetus mature enough to have survived outside the uterus. It is the commonly used term for fetal death, and is used in this thesis to refer to late fetal deaths.

Fetal death: According to the International Classification of Diseases, revision 10 (ICD-10), an early fetal death is death of fetus weighing at least 500 g (or, if birth weight is unavailable, after 22 completed weeks gestation, or with a crown-heel length of 25 cm or more).A late fetal death is defined as a fetal death weighing at least 1,000 g (or a gestational age of 28 completed weeks or a crown-heel length of 35 cm or more).Late fetal deaths are recommended by the World Health Organization as the measure for international comparison of stillbirths.

Early neonatal death: Death of a live born infant within the first seven days.

Neonatal death: Death of a live born infant within the first 28 days.

Post-neonatal death: Death to a live birth after 28 days but within the first year of life.

Infant death: Death in the first year of life.

2) **Mortality**

Stillbirth risk: Number of stillbirths divided by total number of pregnancies (expressed per 1,000).

Early neonatal mortality: Probability of dying during the first seven days of life, expressed per 1,000 live births

Perinatal mortality: Number of perinatal deaths divided by total number of pregnancies (expressed per 1,000).

Neonatal mortality: Probability of dying during the first 28 completed days of life, expressed per 1,000 live births

Post-neonatal mortality: Number of post-neonatal deaths divided by the total number of live births (expressed per 1,000).

Infant mortality: Probability of dying between birth and exactly 1 year of age, expressed per 1,000 live births

Under-five-mortality: Probability of dying between birth and exactly 5 years of age, expressed per 1,000 live births.

3) Parity

Parity: Number of live births for a given woman, counting a multiple birth pregnancy as one.

Nulliparous: No previous live births for a given women.

Multiparous: A woman with one or more previous live births.

4) Births

Live birth: A baby who shows any signs of life after delivery, beyond involuntary gasps (breathing, heartbeat, pulsation of the umbilical cord, or definite movement of voluntary muscles), regardless of whether the umbilical cord has been cut or the placenta delivered.

Preterm birth: A live birth or stillbirth that takes place after 28 but before 37 completed weeks of gestation.

5) Other

Perinatal period: Whereas there is consensus that the perinatal period includes some portion of late pregnancy and some or all the first month of life, this term has been used to refer to at least 10 different periods depending on the time cut offs used and this often causes confusion. In this thesis, we use perinatal deaths to include stillbirths after 28 weeks' gestational age and early neonatal deaths in the first 7 days of life.

Traditional birth attendant: A birth attendant who acquired her skills empirically or through apprenticeship to another traditional birth attendant.

Cluster: One or two villages with most basic social services such as schools, water source, prayer place, market and a minimum population of one thousand (1000) people.

Corridor: One or two villages with most basic social services such as schools, water source, prayer place, market and a minimum population of one thousand (1000) people

Peer support: The provision of emotional, appraisal, and informational assistance by a created social network member who possesses experiential knowledge of a specific behaviour or stressor and similar characteristics as the target population, to address a health-related issue of a potentially or actually stressed focal person

Maternal near miss: A woman who nearly died but survived a complication that occurred during pregnancy, childbirth or within 42 days of termination of pregnancy

Abstract

Universal coverage of good quality facility based care globally could prevent nearly 113,000 maternal deaths, 531,000 stillbirths and 1.3 million neonatal deaths annually by 2020. Yet, only 57% of pregnant Ugandan women choose to deliver at health facilities. This unacceptably low coverage of facility based births could explain, in part, the high maternal and perinatal mortality estimates in Uganda. While multiple studies have examined factors associated with this low utilization of health services around the time of birth, there is inadequate implementation research exploring the best systematic methods that could promote uptake and scale up of facility based births. This study will therefore examine the effect of an intervention package (peer counselling by pregnancy buddies on facility based births, mobile phone messaging promoting facility based births and provision of mama-kits) on the frequency of facility based births and perinatal mortality. The study, a cluster randomized community based intervention trial in post-conflict Northern Uganda, will provide data crucial in framing national policy regarding measures to promote the use of health facilities.

Introduction

In the last two decades, progress in maternal and newborn survival in Uganda has been extremely slow. During this time, maternal mortality reduced by 13% (from 505/100,000 live births to 438/100,000 live births) while neonatal mortality reduced by 21% (from 34/1000 live births to 27/1000 live births) [1]. It is estimated that nearly 46% of all these maternal deaths and 40% of all stillbirths and neonatal deaths happen during labour and on the day of birth [2]. These estimates underline the need for good quality maternal and newborn care around the time of birth [2]. This realization is the basis for the increasing advocacy for facility based births [3]. Universal coverage of good quality facility based care globally could prevent nearly 113,000 maternal deaths, 531,000 stillbirths and 1.3 million neonatal deaths annually by 2020 [4]. Yet, only 57% of pregnant Ugandan women choose to deliver at a health facilities [1] with this proportion dropping in rural areas. Decisions on place of delivery are multi-dimensional, complex and often involve more than one individual. To understand drivers behind these place of delivery decisions, Thaddeus and Maine's three delay model is crucial [1, 5, 6]. This model postulates three delays: 1) delay in deciding to seek care on the part of the individual, the family or both, 2) delay in reaching an appropriate health care facility and 3) delay in receiving adequate health care at the facility. Factors that may contribute to the first delay include lack of awareness of the time of delivery, lack of awareness of the importance of facility births [7], poor perception as regards the health facilities[8], low maternal education and low social economic status [3, 6]. Factors that may lead to the second delay include residence in rural areas and lack of transport [9, 10]. In Uganda, research has shown that pregnant women continue to make seemingly high risk choices which may be a result of ingrained attitudes, cultural practices and beliefs that pertain to birthing practices [11, 12]. Often times, use of health care facilities is considered a last option because of commonly held perceptions regarding unskilled staff as well as abuse, neglect, or poor treatment in hospital settings.

While multiple studies have examined the association between these barriers and utilization of health facilities around the time of birth, there is inadequate implementation research exploring the best systematic methods that could promote uptake and scale up of facility based births.

Therefore, this study will examine the effect of an integrated intervention on the frequency of facility based births, severe maternal morbidity (maternal near miss), perinatal and neonatal mortality. The intervention package, will aim at addressing the first two of the three delays described above and will include: 1) peer counselling by pregnancy buddies on facility based births, 2) mobile phone messaging promoting facility based births and 3) provision of mama-kits. We now review the evidence for each of these in turn

Peer support for facility based births by pregnancy buddies

The significance of social relationships in health promotion and countering of the first two delays cannot be overemphasized [13]. While the literature on peer support and facility deliveries is scanty, multiple studies have found a beneficial effect on other maternal and early childhood outcomes [13]. Positive associations have been reported between peer support and 1) prenatal care [14] 2) perinatal outcomes [15] and 3) breastfeeding[16, 17]. Peer support may impact health outcomes by influencing the decision making process that underpins the first delay described earlier. It is known that during times of indecision and need, as may occur during pregnancy, women may turn to their social networks for support in response to multiple barriers. At such a time, peer supporters, may provide crucial emotional, appraisal and informational support to these women. Such support could explain the reported improvement in health outcomes by decreasing the barriers to care, reinforcing help-seeking behaviour and aiding self-esteem [13].

Mobile phone messaging for promoting facility based births

It is estimated that nearly 60% of the Ugandan adult population own a mobile phone [18]. Mobile phones may be used to reduce the first two delays by cheaply and timely relaying information promoting facility based births. However, data on the use of mobile phone technology on maternal and newborn outcomes is scanty. A Nigerian study found that mobile phone messaging to pregnant women significantly increased skilled attendance at birth [19].This widespread low cost technology could be instrumental in combatting the high maternal morbidity and mortality through increasing facility based births.

This study will examine the role of mobile phone messaging for promotion of health facility births as part of an integrated intervention package.

Provision of mama kits.

A recent systematic review found that the use of mama-kits (birth kits) during delivery was associated with reduced newborn morbidity, mortality and maternal puerperal sepsis [20, 21]. While mama-kits are an essential part of delivery care in many resource limited countries, they are very often unavailable in many health facilities and mothers may be required to procure them on their own from private pharmacies. This may constitute an additional barrier to accessing health facilities for birth. In this study, mama-kits will be provided to the women as part of an intervention package to promote facility based births.

Problem statement and justification of the study

It is estimated that nearly 46% of maternal deaths and 40% of all stillbirths and neonatal deaths happen during labour and on the day of birth [2]. More than half of all Ugandan women give birth at home, and the majority of maternal and perinatal deaths are associated with these births. This realization is the basis for the increasing advocacy for facility based births [3]. These facts justify the need for good quality maternal and newborn care around the time of birth [2].

Universal coverage of good quality facility based care, globally, could prevent nearly 113,000 maternal deaths, 531,000 stillbirths and 1.3 million neonatal deaths annually by 2020 [4]. Yet, only 57% of pregnant Ugandan women choose to deliver at a health facilities [1] with this proportion dropping in rural areas. Decisions on place of delivery are complex. Factors that may contribute to home delivery include lack of awareness of the time of delivery, lack of awareness of the importance of facility births [7], poor perception as regards the health facilities[8], low maternal education, low social economic status [3, 6], residence in rural areas and lack of transport [9, 10].

There is inadequate implementation research exploring the best systematic methods that could overcome these barriers to health facility based births. We propose to undertake a study that will promote and identify the best methods for scaling up, in an equitable and cost-effective manner of facility based births. We shall compare an integrated intervention package of peer support, mobile phone messaging and provision of mama kits to routine government health services on the frequency of health facility births. The effect of single interventions may be small while a package of interventions may have multiplicative effects that are greater than the sum of individual interventions. Our findings of a low cost intervention could be transformed into policy promoting facility based births in Uganda.

Research question

What is the effect of an integrated intervention (peer counselling for facility based births, mobile phone messaging and provision of mama kits) on the frequency of facility based births in Northern Uganda?

Objectives

General Objective

To measure the effect of an integrated intervention on the frequency of facility based births and perinatal outcomes in Northern Uganda

Primary Objective

1) To measure the effect of an integrated intervention on the frequency of facility based births
2) To determine the maternal and perinatal outcomes in South Sudan

Secondary Objectives

1) To measure the effect of an integrated intervention on the perinatal mortality
2) To measure the effect of an integrated intervention on the neonatal mortality
3) To evaluate the acceptability of the intervention by stakeholders (community, health care workers, district administrators)
4) To describe factors associated with maternal and perinatal outcomes in South Sudan
5) To measure the effect of an integrated intervention on optimal breastfeeding (timely breastfeeding (within 1 hour after childbirth) and exclusive breastfeeding)

Conceptual framework

Our framework conceptualizes factors that influence facility based births. These factors are multifactorial and diverse. We integrate the three delays model [22] and the behavioural model of families' use of health services [23]. The three delays model divides these factors into three delays; the delay to decide to seek health care, the delay to get to the health center and the delay in receiving quality care once at the health facility. This is very practical classification especially in low developing settings like Uganda. The behavioural model of families' use of health services divides these factors into; perceived need, predisposition to receive medical care and availability of health care. We incorporated both these models to develop our conceptual frame work which put the social cultural context of the mother, perceived benefit and perception of quality of life under delay one, physical and economical accessibility under delay two and facility based limitations, use of antenatal services and quality of emergency obstetric care under delay 3.

Figure 1: Conceptual framework for factors influencing facility based births

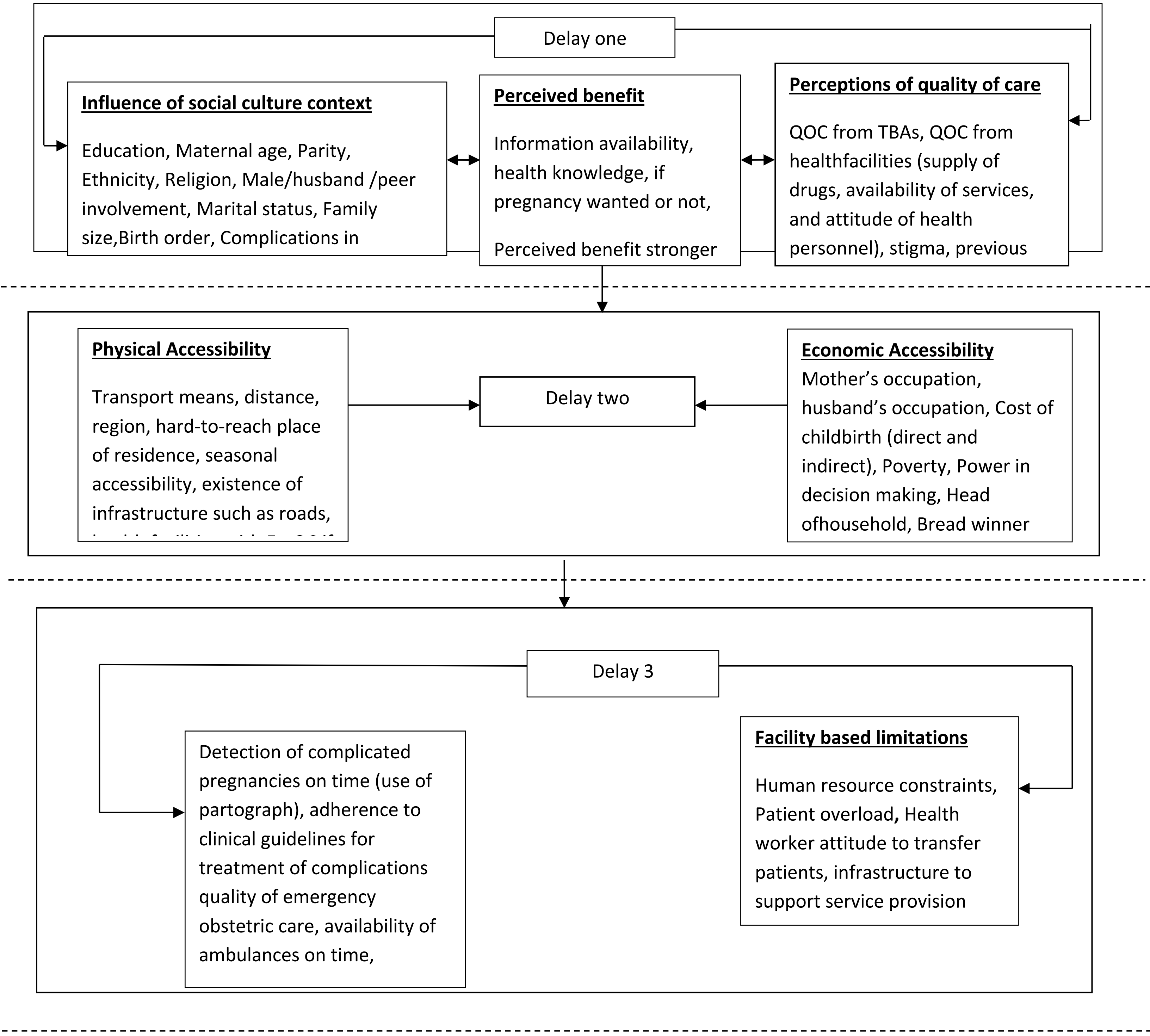

Methods

Study design

In Uganda: This is a community based cluster randomized intervention trial. Clusters (unit of randomization) will be made up of 3 or 4 villages with a population of at least 1000 people. Care will be taken to allow for corridors in between clusters.

In South Sudan: Both community and health facility based cross sectional studies will be carried out to describe the maternal and perinatal outcomes in South Sudan.

Study site

Uganda: The study will be conducted in Lira district, a post conflict area, located in Lango sub-region, Northern Uganda approximately 350km from the capital city, Kampala. It is bordered by Pader in the North, Otuke in the North East, Alebtong in the East, Dokolo in the south and Apac in the West. It covers approximately 1286 square kilometers of land surface. The population of about 403,100 people is predominantly Langi, the main language is Luo and most of the people are peasant farmers. Lira Municipality is the administrative centre of Lira District . Lira district is divided into 3 constituencies; Lira municipality, Erute north and Erute south. It is composed of 13 sub-counties, the biggest of which is Amach.

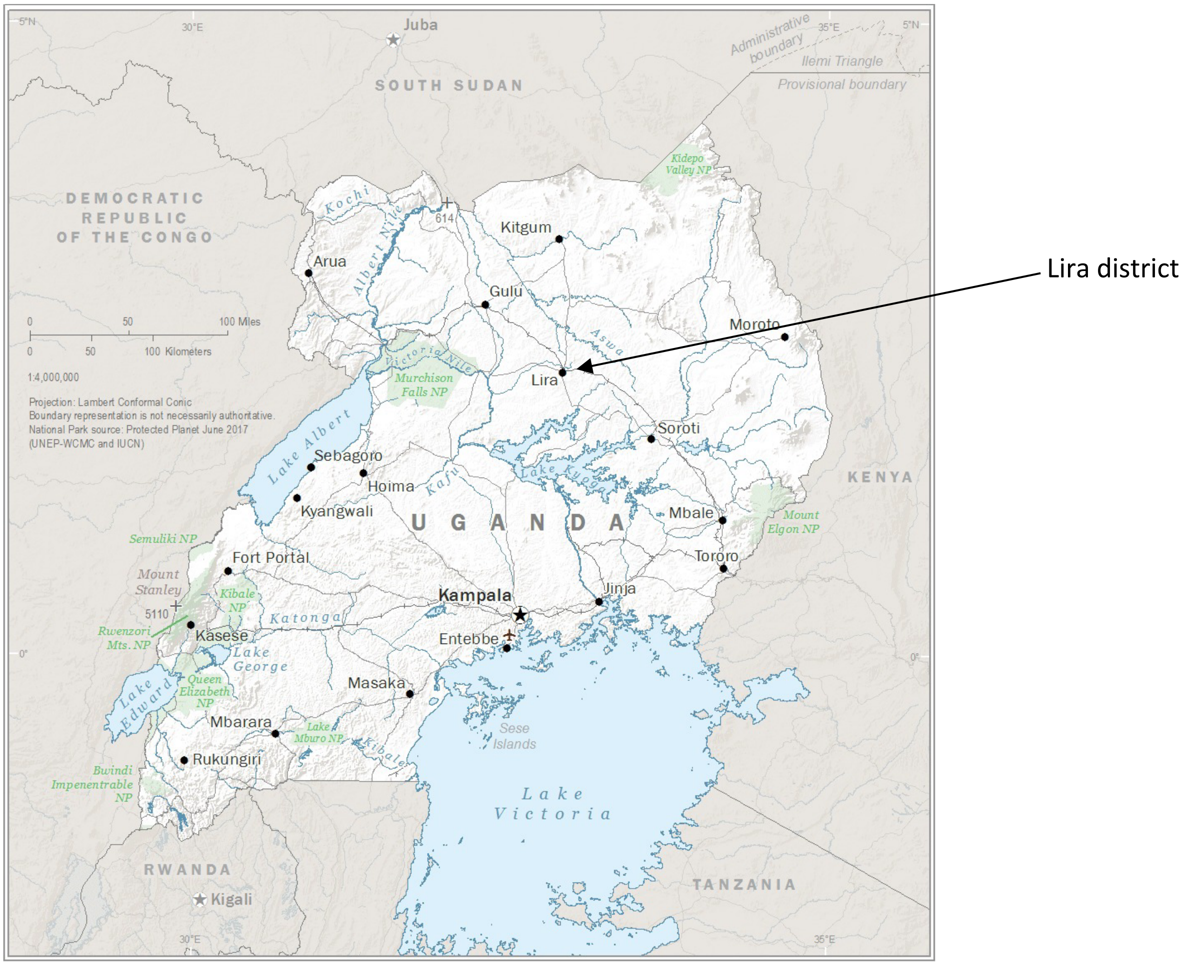

Figure 1: Map of Uganda showing the location of Lira district

South Sudan: Formative research will be conducted in the Central Equatoria state.

Participants

Target population

Pregnant mothers in Uganda.

Accessible population

Pregnant mothers Lira district.

Study population

Pregnant Mothers resident in the study area that will consent to participate in the study

Inclusion criteria and exclusion criteria

Inclusion criteria: All pregnant women at 28 or more weeks of gestation, resident in the selected clusters that consent to participate in the study.

Exclusion criteria: Pregnant women with intention to move from the study area within one year, as well as mothers with psychiatric ailments that prevent them from providing an informed consent will be excluded. All identified pregnancies between 12 and 28 weeks of gestation that aborted or interrupted their pregnancies.

Recruitment

Each cluster will have a recruiter ("pregnancy monitor") who will be a mature woman of good repute living within the cluster. The pregnancy monitors will inform the research centre about pregnant women that may be eligible to participate in the study. A research assistant will be sent to the given cluster to ascertain eligibility, get consent to participate in the study and conduct the recruitment interview.

The Intervention

The intervention will be an integrated package consisting of peer support for facility based births by pregnancy buddies, provision of mama kits and mobile phone messaging. These components will all aim at mitigating the three delays and increasing the proportion of facility based births.

Peer support for facility based births by pregnancy buddies/peer supporters

Peer supporters will be identified and selected from the 19 clusters randomised to peer peer support intervention. These will be women in the reproductive age group (25-45 years) and will be provided with approximately 20 hours of training. Approximately 30 peer supporters will be trained (about double the number needed), to allow for attrition, sickness, etc. After the informed consent, at least three visits will be scheduled for the mothers assigned to peer support by the peer supporter. The first visit will be immediately after recruitment. Counselling by peer supporters will take place during these home visits. These visits will be arranged at the convenience of the mother. Peer supporters will provide substantial information about pregnancy

and child birth and encouragement to deliver at health facilities. Trained in listening, they will address specific concerns of the mothers, correct misinformation, and do these without making mothers feel defensive or inadequate. Peer supporters will work with the women to plan for a health facility delivery (birth preparedness plan) in order to mitigate the physical and economic barriers that contribute to the first two delays. They will encourage the pregnant women to plan and set aside funds for transport to the health facility and for other necessities such as warm baby clothes.   They will deliver messages on the advantages of facility based births, provide simple technical information for overcoming constraints, and provide reminders and encouragement to deliver at health facilities. Peer supporters will not only target the pregnant women, but also husbands, mothers-in-law and other adults in the household who influence birth decisions. The extra visits will be synchronized with the mobile phone personalized information, covering only women with no access to the mobile phones.

Mama Kits

This is the second component of the intervention package. All women in the intervention clusters will be provided with a mama kit consisting of clean plastic sheets, a razor blade, cord ligatures, swabs, two pairs of gloves, cotton and gauze. These kits will be assembled within the community by community groups/pregnancy buddies. They will them be given to the women during the third trimester. The distribution of mama kits will motivate women for health facility births.

Mobile phone messaging

SMS text messages, is the third component of the intervention, will be sent to phone numbers provided by the pregnant women with the overarching aim of increasing facility based births. These messages will convey simple health education messages concerning child birth, expected date of delivery reminders, and encouragement to prepare for and deliver at health facilities. The messages, will be unidirectional, from the research centre to the participants. The text messaging system will be automated and messages will be delivered according to the gestational age of the mother. Relevant data on gestational age will be collected at the first recruitment visit. Text

message content will target the pregnant women, their husbands and mothers- in-law in the local language (Langi).

Sample size

Based on power of 90%, 95% confidence intervals, frequency of home births of 60% in control clusters (UDHS 2006), a 30% reduction in home births (hence an increase in facility births) in intervention clusters, an intraclass correlation coefficient (ICC) of 0.09 (Pagel, 2011), and an average cluster size of 50 pregnant women, approximately 1800 pregnant women in 38 clusters will be required for this outcome

Randomization

The clusters selected for randomization will be chosen in a way that each cluster represents a social and administrative unit. This will minimize interaction between (pregnant) women in intervention and control clusters, and decrease the risk of contamination. Random allocation will be done by individuals not participating in the implementation of the trial. After the 38 clusters are identified, a list of relevant characteristics (such as population size, proximity to health facilities, roads, schools markets and trading centers) will be obtained. Clusters with similar characteristics will be paired and then randomized with the first of each matched pair to Survival pluss integrated intervention or no intervention

Masking

Ascertainment of outcome will be done by research assistants (data collectors) not involved in the exposure allocation. These will be blinded as much as possible to the random allocation of the clusters. This being a behavioral change intervention, it will be difficult to blind the mothers and the investigators to the allocation of the intervention.

Measurements

Primary outcome

The primary outcome will be delivery at a health facility.

Secondary outcomes

Secondary outcomes in this study will be perinatal death and neonatal death. Perinatal death will be defined as pregnancy loss occurring after 28 weeks of gestation (still birth) or death within the first seven days of delivery of a live born child (early neonatal death). Neonatal death will be defined as death of a live born infant within the first 28 days.

Baseline characteristics and potential confounders

Individual level confounders: Data will be collected on several potential individual confounders during pregnancy and immediately after birth. These will include: maternal age, maternal HIV status, parity, maternal education, occupation, past gynaecologic and obstetric history, antenatal care attendance, maternal morbidity, iron supplementation during pregnancy, wealth, household size, tetanus toxoid coverage, health seeking behaviour, singleton or multiple birth, sex of the infant, birth weight, gestational age, place of birth for children, marital status, birth attendant,.

Contextual level confounders: We shall collect data on group/contextual level factors such as presence of electricity in the community, proximity of the clusters to main roads, residence (rural vs. urban) and number and level of health facilities.

Infant feeding practices

The 24 hour recall of a list of food items, the weekly recall of a list of food items and the ever recall will be used to collect infant feeding practices at week 1 and week 6.

Anthropometric measurements

Birth weight, and weight and length of infants at 1 and 6 weeks of age. Weight and height of mother will be measured at 6 weeks post-partum.

Morbidity and mortality

Morbidity (passive surveillance) for diarrhoea, ARI and hospitalisation episodes and mortality through the verbal autopsy.

Data collection and Management

Data collection

All data will be collected using standardized questionnaires and forms for consistency. Data collectors will be trained on all the questionnaires for 1 week before study implementation. At least one senior scientist/medical officer will be employed by the project as a study coordinator to supervise the field team and ensure good quality data. Data will be collected electronically. Questionnaires designed on a desktop PC will be downloaded onto the mobile electronic data collection tools. The interviewers will enter the data directly on the electronic tools. These tools will have an instant check for validity, which does not allow certain types of erroneous responses to be entered. Equipped with GPS, the geographical position of each interview will be recorded.

All mothers will be interviewed at recruitment to establish eligibility and get informed consent, and to obtain data on possible confounding factors as well as descriptive statistics. They will be interviewed again at the end of weeks 1, 2 and 7 after birth to determine the place of birth, pregnancy outcome, vital status of the child and mother and early infant illness (see table below)

Infant feeding patterns will be established through food frequency recalls. The 24 hour recall of breast milk and a list of food items that could have been introduced to the baby with frequency (no. of feeds per day). Weekly food recall with average number of times breast fed or given a food item (from a list of food items). Ever recall for a list of food items including the first time a particular item was introduced.

Data on neonatal illness will be collected through repeated cross sectional surveys at the end of weeks 1, 2 and 7.

Focus of data collection
**Recruitment interview: 2nd or 3rd trimester of pregnancy**
Background characteristics, socio-demographic data on potential individual and contextual level confounders and effect measure modifiers, care seeking practices, cord care and other neonatal care practices
**Follow up interviews: weeks 1, 2 and 7 after birth**

Place of delivery, infant feeding / breastfeeding (initiation and exclusivity), vital status (perinatal and neonatal death), neonatal illness, care seeking practices

Both perinatal and neonatal mortality will data will be obtained using a standard World Health Organization (WHO) verbal autopsy questionnaire that has been validated in Uganda. This questionnaire has both an open ended section for reporting verbatim and a close ended section with filter questions. These questionnaires will be administered as soon as it is socially acceptable.

Data management

Data will be collected electronically on hand held devices in the field. Collected data will be temporarily stored in a secure and encrypted database on a memory card on the handheld and automatically uploaded to the site-office database on a daily basis. The data stored on the handheld computer will be encrypted and protected with a strong password and username combination. The site office will be secured with appropriate measures to prevent theft and unwanted access to hardware and data. Access to collected data at the site will be strictly controlled by the onsite data manager. This security will be possible through the use of industry standard SQL databases i.e. MS SQL Server or MSDE (Microsoft Desktop Engine) that have high level of reliability and built-in security. Strict daily and weekly backup procedures will be observed.

Quality control

The research assistants speaking the local language will be recruited from the study area. They will under-go training on the study objectives, electronic data collection and anthropometry. Steps will be taken to avoid visits by the peer counselor coinciding with visits of the research assistants.The questionnaires will be translated into Langi and back translated into English. The questionnaires will be pre-tested prior and piloted prior to the study. Standard operating procedures (SOPs) will be developed prior to study implementation of the study.Weighing scales and height boards, recommended by UNICEF and WHO, will be zeroed before each field visit

and before each measurement is taken. They will be checked for accuracy daily with known standard weights.

A data manager will be trained in database management. He/she will support the interviewers on electronic data capture. Range and consistency checks will be incorporated in the data collection system to minimize erors. Data will be checked for completeness daily. Standard operating procedures (SOP's) will be developed for each stage of data collection, handling and analysis. These will include data validation, methods for data cleaning and quality control. Support will be ensured through using various communication channels, including online systems, between the technical advisors / site coordinators and field staff. This will enable ongoing capacity development of field staff and problems with hardware or software to be dealt with rapidly and thereby avoiding data loss.

Statistical methods

Data will be transferred from the handheld to the PC and will be analyzed using Stata version 8.0. Continuous variables will be summarized into means, median, and standard deviation. Categorical variables will be summarized into proportions. The results will be presented in form of tables and figures. Relative Risks, Prevalence Ratios, Attributable Risk Reduction, and Number Needed to Treat (support) will be estimated. Baseline characteristics of the mother, household and new born will be compared between intervention and control groups to check for group comparability and identify potential confounders. Descriptive statistics for timing, frequency and coverage of the intervention will be computed. Analysis for the main outcome will be based on intention-to-treat. Therefore, all mother-infants enrolled into the study will be included in the final analysis regardless of whether they received the allocated intervention or not. The risk of having a home birth in the intervention group will be compared to a similar risk in the control group using binomial regression with a log link. Multivariable analysis will be used to take into account confounding. Only potential confounders associated with the outcome with a P-value of <0.025 or whose inclusion in the model results in a 10% in the beta estimate of the main outcome will be left in the final model. Secondary outcomes will also be analysed in a similar manner.

Equity impact analysis: Further secondary analysis will compare the effectiveness of the intervention across strata of education levels, wealth quintiles and residence in order to assess the impact of the intervention on health and economic equity. All the data required for this analysis will be collected prospectively and if the sample size allows, we shall estimate the effects of focussing this intervention on the most vulnerable groups. The concentration index, a bivariate measure of the distribution of variables such as coverage of the intervention will be used to measure improved equity in outcomes.

Economic evaluation: This will involve measuring the cost of the intervention, estimating costs of and economic benefits from increased facility births and reduced perinatal deaths, and cost-effectiveness analyses using standard methods. The ingredients approach, which combines data from interviews with program managers, facility records and other relevant sources will be used to cost the intervention and the control. This method captures direct, indirect health care provider, recurrent and indirect costs. During our household data collection visit, data will be collected on costs for the study participants. If the intervention is effective, DALYs averted from it will be calculated.

Derivative research: We recognise that through this study, a cohort with a rich database will be generated. We shall plan from the very beginning for long term follow- up and when additional funding is available in the future, encourage ethical use of this data to explore other epidemiologic questions of public health importance. For example, questions that could be examined in the future with additional funding include: the effects of prenatal and early life exposures on cognitive development or non-communicable diseases in later childhood and adulthood.

Ethical issues

Institutional permission to conduct the study will be obtained from the Faculty of Medicine, Makerere University; Uganda National Council of Science and Technology; Ministry of Health and Lira Local Government. Individual written consent will be obtained from the respondents in the study. The consent process will explain the nature of the study, the risks and benefits of participating in the study, the intervention and that allocation is by a random process.

Confidentiality of information and the right of the respondent to withdraw from the study at any time during the study will be explained to the participants. Research assistants will be trained on confidentiality.

Dissemination of findings

Relevant staff will participate in discussing and drawing conclusions from the completed data analyses. Internal guidelines for authorship have already been agreed upon. Key staff will submit and present abstracts to relevant national, regional and international scientific and policy-making meetings. The consortium will disseminate the results through abstracts in meeting proceedings, scientific publications in international refereed journals, and prepared presentations for relevant authorities and policy makers.

References

1. **Uganda Bureau of Statistics (UBOS): Uganda Demographic and Health Survey** In. Edited by UBOS Kampala U, and ICF International Inc.; Calverton, MD, USA: 2012. ICF International Inc; ; 2011: 1-257.
2. Lawn JE, Blencowe H, Oza S, You D, Lee AC, Waiswa P, Lalli M, Bhutta Z, Barros AJ, Christian P *et al*: **Every Newborn: progress, priorities, and potential beyond survival**. *Lancet* 2014, **384**(9938):189-205.
3. Samarasekera U, Horton R: **The world we want for every newborn child**. *Lancet* 2014, **384**(9938):107-109.
4. Bhutta ZA, Das JK, Bahl R, Lawn JE, Salam RA, Paul VK, Sankar MJ, Blencowe H, Rizvi A, Chou VB *et al*: **Can available interventions end preventable deaths in mothers, newborn babies, and stillbirths, and at what cost?***Lancet* 2014, **384**(9940):347-370.
5. Thaddeus S, Maine D: **Too Far to Walk - Maternal Mortality in Context**. *Social science & medicine* 1994, **38**(8):1091-1110.
6. Bhutta ZA, Das JK, Bahl R, Lawn JE, Salam RA, Paul VK, Sankar MJ, Blencowe H, Rizvi A, Chou VB *et al*: **Can Available Interventions End Preventable Deaths in Mothers, Newborn Babies, and Stillbirths, and at What Cost? EDITORIAL COMMENT**. *Obstet Gynecol Surv* 2014, **69**(11):641-643.
7. Titaley CR, Hunter CL, Dibley MJ, Heywood P: **Why do some women still prefer traditional birth attendants and home delivery?: a qualitative study on delivery care services in West Java Province, Indonesia**. *BMC Pregnancy Childbirth* 2010, **10**(43):1471-2393.
8. Oyerinde K, Harding Y, Amara P, Garbrah-Aidoo N, Kanu R, Oulare M, Shoo R, Daoh K: **A qualitative evaluation of the choice of traditional birth attendants for maternity care in 2008 Sierra Leone: implications for universal skilled attendance at delivery**. *Matern Child Health J* 2013, **17**(5):862-868.
9. Exavery A, Kante AM, Njozi M, Tani K, Doctor HV, Hingora A, Phillips JF: **Access to institutional delivery care and reasons for home delivery in three districts of Tanzania**. *Int J Equity Health* 2014, **13**(48):1475-9276.
10. Tey NP, Lai SL: **Correlates of and barriers to the utilization of health services for delivery in South Asia and Sub-Saharan Africa**. *ScientificWorldJournal* 2013, **28**(423403).
11. Anyait A, Mukanga D, Oundo GB, Nuwaha F: **Predictors for health facility delivery in Busia district of Uganda: a cross sectional study**. *BMC pregnancy and childbirth* 2012, **12**.
12. Moyer CA, Mustafa A: **Drivers and deterrents of facility delivery in sub-Saharan Africa: a systematic review**. *Reproductive health* 2013, **10**:40.
13. Dennis CL: **Peer support within a health care context: a concept analysis**. *International journal of nursing studies* 2003, **40**(3):321-332.
14. Lapierre J, Perreault M, Goulet C: **Prenatal peer counseling: an answer to the persistent difficulties with prenatal care for low-income women**. *Public health nursing* 1995, **12**(1):53-60.
15. Heins HC, Jr., Nance NW, Ferguson JE: **Social support in improving perinatal outcome: the Resource Mothers Program**. *Obstetrics and gynecology* 1987, **70**(2):263-266.

16. Dennis CL, Hodnett E, Gallop R, Chalmers B: **The effect of peer support on breast-feeding duration among primiparous women: a randomized controlled trial**. *CMAJ : Canadian Medical Association journal = journal de l'Association medicale canadienne* 2002, **166**(1):21-28.
17. Tylleskar T, Jackson D, Meda N, Engebretsen IM, Chopra M, Diallo AH, Doherty T, Ekstrom EC, Fadnes LT, Goga A *et al*: **Exclusive breastfeeding promotion by peer counsellors in sub-Saharan Africa (PROMISE-EBF): a cluster-randomised trial**. *Lancet* 2011, **378**(9789):420-427.
18. Uganda MoH: **UGANDA AIDS INDICATOR SURVEY 2011**. In. Kampala, Uganda: Ministry of Health, Uganda; 2012.
19. Oyeyemi SO, Wynn R: **Giving cell phones to pregnant women and improving services may increase primary health facility utilization: a case-control study of a Nigerian project**. *Reproductive health* 2014, **11**.
20. Hundley VA, Avan BI, Braunholtz D, Fitzmaurice AE, Graham WJ: **Lessons regarding the use of birth kits in low resource countries**. *Midwifery* 2011, **27**(6):e222-e230.
21. Seward N, Osrin D, Li L, Costello A, Pulkki-Brännström A-M, Houweling TA, Morrison J, Nair N, Tripathy P, Azad K: **Association between clean delivery kit use, clean delivery practices, and neonatal survival: pooled analysis of data from three sites in South Asia**. *PLoS medicine* 2012, **9**(2):e1001180.
22. Thaddeus S, Maine D: **Too far to walk: maternal mortality in context**. *Social science & medicine* 1994, **38**(8):1091-1110.
23. Andersen R: **A behavioral model of families' use of health services**. *Research Ser* 1968(25).

Appendices

Consent forms

RESEARCH PARTICIPANT RECRUITMENT INFORMED CONSENT DOCUMENT

Protocol Title: SURVIVAL PLUSS: Increasing capacity for mama-baby survival in post-conflict Uganda and South Sudan

This study is being done by doctors from Makerere University in Uganda, and from the University of Bergen in Norway.

Principle Investigator: Prof. James K Tumwine

Co-Investigators:
Prof Grace Ndeezi,
Dr.Victoria Nankabirwa,
Prof Thorkild Tylleskar
Dr Elizabeth Bwanga
Prof. Paul Waako, Busitema University
Dr. Joyce Kaducu, Gulu University
Dr. Jino David, University of Juba, South Sudan
Prof Sebit Boy, University of Juba, South Sudan

I. Background

This form gives you information about a research study that will also be discussed with you. You have been asked to join this study because you are pregnant. This study is designed to find out the effectiveness of an intervention to promote use of health facilities. Once you have all your questions answered about the study, and if you agree to join the study, you will be asked to sign this form.

You are free to either join or not join the study and even when you have joined, you are free to leave the study at any time. You will not lose any benefits by not joining the study and you will receive the standard of care.

.

II. Study procedures

You will be interviewed before pregnancy and after the birth of your baby. Your height and weight will also be measured.

III. Risks

There are no major risks involved in this study.

IV. Benefits
There will be monitoring of you and your baby till 6 weeks after birth.Other than that, there will be no other direct benefits to you from this study; however the findings may help us to improve the health of mothers and babies in Uganda.

V. Alternative treatments
Joining this study is voluntary; you do not have to join the study if you do not want to. Refusal to join the study will not place you or your child at risk of losing access to medical care. If you choose to not join the study or if you decide to leave the study, you will continue to receive the exact treatment offered by the responsible authorities.

VI. Confidentiality
Personal information will be collected about you and your child, and it will be kept strictly in confidence, under lock and key. Only the authorized study personnel will have access to you and your child's medical records and personal identifiers.

VI. Costs
There will be no cost to you and/or your child for the study process.

VII. Questions and queries
If you ever have questions about this study, need any more information, or if you have a health problem or are hurt in the study, you should contact study coordinator, Dr. ………………at 07……………or Dr. Victoria Nankabirwa. at telephone number 0755757460.
If you have questions about your rights as a research volunteer, you may contact, the School of Medicine Research and Ethics Committee (SOMREC) through its deputy chairman Dr. Imelda Namagembe on telephone number 0772404902
.
The study staff will be happy to help you contact the right person to answer any questions you have.

Your signature or thumbprint below means that you understand the information given to you about the study and in this consent form. If you sign or place your thumb print on this form, it means that you agree that you and your baby will take part in the study. You are not giving up any of your and your child's legal rights by signing this informed consent document.

WE WILL OFFER YOU A COPY OF THIS CONSENT FORM.
Do not sign after the expiration date of: _______________________

NOT VALID WITHOUT THE IRB STAMP OF CERTIFICATION

STATEMENT OF CONSENT:
I have read (or someone has read and explained to me) the information in this consent form. I understand why this study is being done, what will be done, and the risks and benefits as described in this written summary. I agree by my own choice to take part in the screening for this research study.

______________________________	______________________________	________________
Participant's Name (print)	Participant's Signature or Thumb Print	Date

<u>For illiterate volunteers</u>: I confirm that the information contained in this written consent form has been read and explained to the participant. She has to the best of my knowledge understood the purpose, procedures, risks and benefits of screening for the study and has voluntarily accepted to have her child take part in the study.

<u>For those placing thumbprint only</u>: I attest that the participant who states that her name is

____ has placed her thumbprint on this

consent form of her own free will on this day ______________________________.

______________________________	______________________________	________________
Name of Witness to Consent Process (print)	Witness' Signature	Date

<u>For all volunteers:</u> I have explained the purpose of this study to the volunteer and have answered all of her questions. To the best of my knowledge, she understands the purpose, procedures, risks and benefits of this study.

______________________________	______________________________	________________
Name of person obtaining consent (Print)	Signature of person obtaining consent	Date

Papara me yee

PAPARA A JO ME ONIANG DANG OYE DONYO I AKWEDA

Nying kwan: Kwan me ngiyo kwo a jo: Ngiyo kit ame tic kede yor apol (rok wii, cim cing, mama kit) i kom nywal ame timere i kumalo me Uganda.

Otim Kwan man obedo dakata ame yaa i Makerere University i Uganda kede eno kene yaa i University me Bergen i Norway.

Akwed lok a dwong: Prof. James K Tumwine

Olub kor okwed lok: Prof Grace Ndeezi
Dr.Victoria Nankabirwa,
Prof Thorkild Tylleskar,
Dr. Elizabeth Bwanga,
Prof. Paul Waako,
Dr. Joyce Kaducu,
Dr. Jino Meleby,
Prof. Boy Sebit

Jo ame note kede okwed lok:
Dr. Frederick Froen,
Dr. Jagrati Jani,
Prof. Halvor Sommerfelt

I. Nge lok

Papara man tye me miyo ngec I kom akweda kwan ame dang obino leyo kedi. Okwai me donyo I kwan man pi en yin i yac. Kwan man oyiko me nwongo teko me kony me cwako tic kede jami me yot kom. Ka dong odok iyi apenyi ducu i kom kwan, kede ka iyee donyo I kwan man, obino kwayi me keto cingi I papara man. Itye agonya me donyo onyo pe donyo i kwan man dang kadi bedi idonyo, itye agonya me ya oko ikwan man icawa moro keken. Pe ibino rwenyo magoba moro keken ka pe idonyo ikwan ento pwod ibino nwongo gwok ena jwi.

II. Yore me kwan

Obino penyi apwod per u iyac kede i yonge nywalere atini, bori kede peki obino dang pimo.

III. Peki

Petye peki adongo ame kwako kwan man

IV. Magoba

Obino neni kede atini pi cabite abicel iyonge nywal. Apat imanono, petye magoba moro atir atir boti ikwan man; ento adwogi mere room konyo wa iyiko yot kom toto kede otino me Uganda.

V.Kony apat

Donyo ikwan man tye imiti angatoro keken, pe myero idony ikwan man k ape imito. Kwero donyo ikwan man pe keti onyo atini ipeko me pe nwongo kony me yot kom. Ka iyero pe me donyo ikwan man onyo itamo me weko kwan man, pwod ibino mede kede nwongo kony ateteni ibot odongo ame tic man kwako gi.

VI. Imung

Obino rayo kop akwako komi kede kom atini, dang te gwoko ateni ite mung, ite pung kede agony. Odongo me kwan man ame cik oyeyi gi aye bino bedo ingec me yot komi wun kede atini kede kop akwako komi keni.

VI. Wel Cul

Petye cul moro keken boti onyo bot atini ikare me kwan man

VII. Apeny kede peny me niang

Ka onyo apenyoro keken tye akwako kwan man, imito ngec okene, onyo itye kede peko iyot komi onyo inwongo cwer cwuny ikwan man, iromo kubere kede adwong kwan, Dr………………………………….……… i 07……………………….onyo dakta me kwan, Dr Victoria Nankabirwa. i namba cim 0755757460

Ka itye kede apeny akwako twero ni acalo akweda odyere, iromo kubere kede the School of Medicine Research and Ethics Committee (SOMREC) kun ibeyo ikom alub won kom Dr. Imelda Namagembe I namba cim 0772404902.

Otic me akweda man cwiny gi abedo nyom me konyi me kubere kede dano atwero gamo apenyoyo keken ame I tye kede.

Keto cingi onyo diyo cingi piny kane nyutu ni iniang ngec ame jo mii akwake kede kwan man kede i papara me yee man. Ka idiyo onyo i keto alama cingi i papara man, tyene gonyere n ii yee ni in kede atini bino bedo i kwan man. Pet ye ni atye a miyo twero ni kede twero atini ame cik yee n ii bed kede ka i keto cingi i papara man me ye came i kwano do kite niang iye.

WA BINO MIYI KOPI ME PAPARA ME YEE MAN.

Pe i ket cingi ka nino dwer man okato: _______________________

PE TYE ATIR KA STAMP ME MIYO TWERO A IRB PE TYE KAN

KOBO YEE:

A kwano (onyo ngatoro okwano te tito bota) ngec ame tye i papara kan. Aniang pi ango oweko jot ye ka timo kwan man, ngo abi timere, kede peki kede magoba kite ame otite kede i coc a cegi. Ayee i miti na kena me bedo i akina jo ame obi yero gi me bedo i kwan mane.

______________________________	______________________________	________________
Nying a kwan (Coo cing adongo)	Keto Cing a kwan onyo Diyo cinge	Nino dwer

<u>Pi dano ame odyere ameme pe ngeyo kwan</u>: A niang ni ateni ngec ame tye i papara me ye man jo kwano dang tye kobo bot akwan. En i tut ta ngeca oniang ngo ma kwan man ocung pire, yore, peki kede magoba me bedo i kwan man dang te ye pire keken ni atine bed i kwan man.

<u>Pi dano ame tye a geyo cingi keken</u>: A neno ni dano ni ame tye a kobo nyinge ni en nyinge tye ____ ogeyo cinge i papara man me yee dayoketo cinge I papara ni kede miti mere ______________________________.

______________________________	______________________________	________________
Nying caden me neno yee man (Coo coc adongo)	Cing caden	Nino dwer

<u>Pi odyere duc:</u> A kobo tek pi kwan man bot jonu yee bedo i kwan man dang en otyeko gamo apeny mere ducu. I nianga atut, en niang tek pi kwan man, peki karael kede magoba a kwan man.

______________________________	______________________________	________________
Nying dano ame tye a yenyo yee (Coo nukta adwong)	Keto cing dano ame tye ka yenyo yee	Nino dwer

Questionnaires

A: Survival PLUSS Recruiment Questionnaire
List of abbreviations
EF = Electronic form
Radio Button (RB), One alternative only
Check Boxes (CB), Multiple alternatives allowed
Other alternatives: text (tx) and numeric (Num) (The rest: See Electronic Form manuals and separate entry SOP)
Langi = Local language

Rule for all categorical answers: DNK tick in under question mark
Rule for all numerical answers: DNK = 99

SECTION 0 Background

Page a (1)

QUESTION ENGLISH	ANSWER ENGLISH	SKIP INSTRUCTION	COLUMN FOR CODING
1. Site			EF: RB
2. Interviewer	__________		EF: RB<br>4 LETTER CODE;<br>UPPER CASE<br>Choose between drop down list/text<br><br>VN: 01a02<br>**VN ALTERNATIVES: KEEP 4 DIGIT CODE**
3. Date:			EF: Date<br>VN: 01a03
4. Time			EF: Time<br>VN: 01a04

5. GPS	1.<br>2.<br>3.	Optional: Can be deleted as an entry alternative for those who will have this automatic in EF<br>Needs a cable in addition to the GPS<br>Alt recorded in m	EF: GPS<br>VN: 01a05 a1:E/(W) ###°##.###<br>a2: N/S ##°##.###<br>a3: #### (#-####)

Enter the following under the Electronic Form type question Label/Title. Remember max. 250 characters.
English: Consent for screening:

READ OUT LOUD:
We come from The Department of Paediatrics and Child Health of Makerere University Medical School. We are conducting a study on Maternal and Child Health. Will you allow me to include you in the study? Thank you so much. Now let's answer the following questions together.

Langi: Consent for screening:
READ OUT LOUD

6. Oral consent for screening given:	1. [_] Yes<br>0. [_] No		[_] Eyo<br>[_] Pee	**If no,<br>Rule EF:<br>No → Discontinuation from SI<br>- Say thank you and ask for reason for non-participation; fill in separate form**	EF: RB<br>VN: 01a06
7.Main language spoken	1. [_] Langi<br>2. [_] Acholi<br>3. [_] Lugbara<br>4. [_] Alur<br>5. [_] Kumam<br>6. [_] Itesot<br>7. [_] Luganda<br>8.[_]English<br>9.Other[_] Specify……….				EF:RB<br>VN: 01a07

8. Which language is the interview translated into:	1. [_] Langi<br>2. [_] Kumam<br>3. [_] Itesot<br>4. [_] Acholi<br>5. [_] Luganda<br>9. [_] Other specify<br>______________				EF: Tx<br>VN: 01a08
9. External translator needed:	1. [_] Yes<br>0. [_] No		[_] Eyo<br>[_] Pee		EF: RB<br>VN: 01a09

	**Do not read out:**<br>10. Note Sub-County/Division	**Do not read out:**<br>11. Note ward/parish= CLUSTER CODE	12. **Read English:** What is the name of your village/cell?<br>**Read Langi:**	**CODING!**
Residence	1. [_] Amach<br>2. [_] Agali<br>3. [_] Adekokwok<br>4. [_] Ngetta<br>5. [_] Ogur<br>6. [_] Agweng<br>7. [_] Lira<br>8. [_] Barr<br>9. [_] Aromo<br>10. [_] Adyel<br>11. [_] Ojwina<br>12. [_] Central<br>13. [_] Railway	1. [_]<br>2. [_]<br>3. [_]<br>4. [_]<br>5. [_]<br>6. [_]<br>7. [_]<br>8. [_]<br>9. [_]<br>10. [_]<br>11. [_]<br>12. [_]<br>13. [_]<br>14. [_]<br>15. [_]<br>16. [_]<br>17. [_]<br>18. [_]<br>19. [_]<br>20. [_]<br>21. [_]<br>22. [_]<br>23. [_]<br>24. [_]	1a. [_]<br>1b. [_]<br>2. [_]<br>3. [_]<br>4. [_]<br>5. [_]<br>6. [_]<br>7. [_]<br>8a. [_]<br>8b. [_]<br>9. [_]<br>10. [_]<br>11a. [_]<br>11b. [_]<br>12a. [_]<br>12b. [_]<br>13. [_]<br>14a. [_]<br>14b. [_]<br>15a. [_]<br>15b. [_]<br>16a. [_]<br>16b. [_]<br>17a. [_]<br>17b. [_]<br>18a. [_]<br>18b. [_]	*EF: RB/ All Mand*<br>*VN: q 10 Subcounty/Division : 03a10*<br>*q 11 Ward/Parish: 03a11*<br>*q 12 Village: 03a12*

			19. [_]<br>20. [_]<br>21a. [_]<br>21b. [_]<br>21c. [_]<br>22a. [_]<br>22b. [_]<br>22c. [_]<br>23a. [_]<br>23b. [_]<br>24a.[_]<br>24b.[_]<br>24c. [_]	

Screening questions:

EF page b (2)

QUESTION ENGLISH	ANSWER ENGLISH	SKIP INSTRUCTION	COLUMN FOR CODING
1. Do you have any intention to move from your village/cell within the next six months?	1. [_] Yes<br>0. [_] No	**SKIP: If no, skip to q. 3**	EF: RB<br><br>VN: 01b01
2. Where will you move to?	1. [_] Within the cluster/village<br>2. [_] Outside the cluster/village	**If alternative 2 chosen Rule EF:<br>No → Discontinuation from SI<br>- Say thank you and fill in form 'reason for non-participation**	EF: RB<br>VN: 01b02
3. I can see / have understood / have been told that you are pregnant now, can you please tell me how many months you have been pregnant?.	1. [_] Seven or more than seven months pregnant, specify Months : ____________ (#)<br>2. [_] Less than seven months pregnant<br>3. [_] Have given birth	**Alternative 2: Enable question 7 by branching<br>Alternative 3: Enable question 8 by branching**	EF: RB<br>a1: Num<br>a2: Nothing<br>a3: Nothing<br>VN: 01b03

Make rule EF: p b q. 2=alt 1, 3=≤7 mo or delivered, 4(-SA)= Yes →Continue
Make rule EF p b q. 1=Yes and q. 2 = alt 2, 3> 7 (ask for permission to come back later), q. 4 = No → Discontinuation

4. All inclusion criteria fulfilled:	1. [_] Yes<br>0. [_] No, specify		[_] Eyo<br>[_] Pee	**Rule EF:**<br>**No →**<br>**Discontinuation from SI**	EF: RB<br>VN: 01b04
6. No exclusion criteria fulfilled	1. [_] Yes<br>0. [_] No, specify		[_] Eyo<br>[_] Pee	**Rule EF:**<br>**No →**<br>**Discontinuation from SI**	EF: RB<br>VN: 01b06

Inclusion criteria:
Lives in the selected cluster
Is pregnant
Has no plans to move outside the cluster within 6months
Exclusion criteria:

7. If < 7 months pregnant : Ask for permission to come back later, and note approximate date of revisit:	dd/mm/yyyy				EF: Date<br>VN: 01b07<br>Disabled, to be enabled see q. 3

PAPER CONSENT FORM EXPLAINED AND ACCEPTED: USI given
If not, ask for reason for non participation and note it down on the form "Reason for non-participation"

9. Participant Id no/ Unique Subject Identifier (USI)	####		EF: Num<br>VN: 01b09<br>4 digit code starting at 1001 all sites
10. Reason for non-participation	_1. [_] form filled	RULE: Do separate form: Reason for non-participation on paper, copy, fill in separately	EF: Tx<br>VN: 01b10

SECTION I Mother's characteristics:

Electronic Form page c (3)

QUESTION ENGLISH	ANSWER ENGLISH	RULE/ SKIP INSTRUCTION	COLUMN FOR CODING
1. How old are you?	_______ *(##)*	**RULE:** Age in completed years.	EF: BIRTH DATE FORM<br><br>VN: 01c01
2. What's your date of birth?	____________(day/month/year)		EF: Date<br>VN: 01c02
3. What is your highest level of education?	0. [_] None<br>1. [_] P1<br>2. [_] P2<br>3. [_] P3<br>4. [_] P4<br>5. [_] P5<br>6. [_] P6<br>7. [_] P7 PLE<br>8. [_] S1<br>9. [_] S2<br>10.[_] S3<br>11.[_] S4 O-level<br>12.[_] S5<br>13.[_] S6 A-level<br>14.[_] Certf 1 yr<br>15.[_] Certf 2 yrs<br>16.[_]Degree/Bach<br>17.[_] > Bachelor<br>18.[_] Other, completed years: __	Comment: Note completed year/level	EF: RB<br>VN: 01c03<br><br>a18: tx
4. Do you have any vocational training or have you had any apprenticeship? (-SA)	1. [_] Yes<br>0. [_] No		EF: RB<br>VN: 01c04
5. Can you read?	1. [_] Yes<br>0. [_] No		EF: RB<br>VN: 01c05
6. Can you write?	1. [_] Yes<br>0. [_] No		EF: RB<br>VN: 01c06
7. Are you single, married, co-habiting, widowed, divorced or separated now?	1.[_] Single ↓<br>2.[_] Married<br>3.[_] Co-habiting<br>4.[_] Widowed<br>5.[_] Divorced/ Separated	**SKIP: If not married (alt.2) skip to q.15**	EF: RB/Mand<br>VN: 01c07

8. How did you get married?	1. [_] Religious<br>2. [_] Civil<br>3. [_] Traditional<br>4.[_] Co-habiting		EF: RB<br>VN: 01c08
9. Does your husband have any other wives?	1. [_] Yes<br>0. [_] No↓<br>3. [_] Do not know↓	**SKIP: If no(alt. 2) or do not know (alt. 3), skip to q.15**	EF: RB<br>VN: 01c09
10. How many?	___ (#(#))		EF: Num<br>VN: 01c10
11. Do you share the same compound?	1. [_] Yes<br>0. [_] No		EF: RB<br>VN: 01c11
12. What is your tribe?	1. [_] Langi<br>2. [_] Acholi<br>3. [_] Lugbara<br>4. [_] Alur<br>5. [_] Kumam<br>6. [_] Itesot<br>7. [_] Luganda 8.[_]English<br>9.Other [_]<br>Specify……….		EF: RB<br>VN: 01c12<br>Overall VN in Uganda is 01c15U<br>a1=501<br>The other countries replace a1=501 with local lists and name it 40x,60x
13. What is your religion?	1. [_] Protestantism/ National Church<br>2. [_] Catholisism<br>3. [_] Islam<br>4. [_] Hinduism<br>5. [_] Buddhism<br>6. [_] Judaism<br>7. [_] Jehovas witness/ mormones<br>8. [_] SDA<br>9. [_] Traditional religion<br>10. [_] Other, specify<br>_________________	Protestantism=<br>Any national church or free church sharing the basic theological concepts with Protestantism as Anglicans/ Lutherans/ Calvinists/ Baptists/ Methodists/ Pentecostals/<br>Newer free churches etc.<br>SDA: Seventh Day Adventists	EF: RB<br>VN: 01c13
14.On what date did you have the last delivery	……/……../………..		EF:RB<br>VN:01c14

15.Mode of delivery?	1. [_] Spontaneous Vaginal delivery<br>2. [_] Assisted Vaginal delivery<br>3. [_] Caeserian Section		EF:RB<br>VN:01c15
16.Is the baby ?	1. [_] Singlet<br>2. [_] Twin<br>3. [_] Triplet<br>4. [_] other specify<br>______________________	In case of twins or triplets use a separate questionnaire?	EF:RB<br>VN:01c16
17. Is the baby alive?	1. [_] Yes<br>0. [_] No		EF:RB<br>VN:01c17
18. Baby's sex?	1. [_] Male<br>2. [_] Female		EF:RB<br>VN:01c18
19.Age of baby in months	________ (Months)		EF:RB<br>VN:01c19
20.What is the highest level of school that your husband attended?	0. [_] None<br>1. [_] P1<br>2. [_] P2<br>3. [_] P3<br>4. [_] P4<br>5. [_] P5<br>6. [_] P6<br>7. [_] P7 PLE<br>8. [_] S1<br>9. [_] S2<br>10.[_] S3<br>11.[_] S4 O-level<br>12.[_] S5<br>13.[_] S6 A-level<br>14.[_] Certf 1 yr<br>15.[_] Certf 2 yrs<br>16.[_]Degree/Bach<br>17.[_] > Bachelor<br>18.[_] Other, completed years: __		EF:RB<br>VN:01c20
21.What is the primary(main) occupation of your husband?	[_] salaried worker<br>[_] Bussinessman<br>[_] labourer<br>[_] Farmer<br>[_] Others(specify)……..		EF:RB<br>VN:01c21

SECTION II Pregnancy history:

Electronic Form p d (4)

**QUESTION ENGLISH**	**ANSWER ENGLISH**	**RULE/ SKIP INSTRUCTION**	**COLUMN FOR CODING**
1. How many children have you given birth to?	_______ *(#(#))* *!	**ALT 2: See skip instruction SIII and SVII if alternative 2 ticked off**	EF: Num<br>VN: 02d01
Now I will ask you questions about the child you expect:<br>2. Can you please tell us when your last menstrual period started?	___/____/______<br>dd/mm/yyyy	**To be disabled and activated if question** : 01b03 alternative 3 is ticked off<br><br>**PROBE: If she does not know**	EF:<br><br>VN: 02d02<br>The probing questions fits into the birthday card and we also see a calculated GA
**PROBE:**<br>Maybe you remember which month it was?<br>Maybe you remember if it was in the beginning, middle or end of that month?			
3. Do you have any card from the ante natal clinic (ANC-card)?	1. [_] Yes<br>0. [_] No ↓	**SKIP: If no, skip to S III**	EF: RB<br>VN: 02d03
4. May I please see it?	1) Note last menstrual period given in the card:<br>____/____/______<br><br>Note estimated duration of pregnancy at a given date:<br>2) Duration in months ______<br>3) Given date:<br>____/____/______<br>dd/mm/yyyy<br><br>4) Note estimated date of delivery:<br>____/____/______<br>dd/mm/yyyy		EF: CB<br>a1) Date<br>a2) Num<br>a3) Date<br>a4) Date<br><br>VN: 02d04

SECTION III Health facility delivery experience and intentions:
SKIP instruction: If section II, question 1 = Alternative 2 Skip to question 5

English:
Now I am going to ask you questions about the children you had before the one you are expecting now (*if she has already given birth;* before the last one you gave birth to less than one week ago).
Langi:
Electronic Form p. e (5)

QUESTION ENGLISH	ANSWER ENGLISH	RULE/ SKIP INSTRUCTION	COLUMN FOR CODING
1. Have you ever delivered any of your children in a health facility?	1. [_] Yes<br>2. [_] No<br>3. [_] INAP	**SKIP: If No, skip to q. 3**	EF: RB<br>VN: 03e01
2. How many children have you delivered in a health facility?	______		EF: Num<br>VN: 03e02
3. How would you rate your health facility experience for the last pregnancy?	ANSWER GIVEN IN:<br>[_]Very good<br>[_] Good<br>3. [_] Fair<br>4. [_] Bad		EF: RB<br>VN:03e03
4. have you/or someone very close to you ever delivered from a TBA?	1. [_]Yes<br>2. [_]No<br>1. [_] Don't know		EF: RB<br>VN:03e04
5.How would you rate their services?	1. [_]Good<br>2. [_]Fair<br>3. [_]Bad		EF: RB<br>VN:03e05
6.How would you compare health facility to TBA in terms of quality of care during birth?	1.Better<br>2.Worse<br>3.Same		EF: RB<br>VN:03e06
7.How would you describe your experience at your last health facility visit during pregnancy or childbirth?	1. [_]very comfortable<br>2. [_]somewhat comfortable<br>3. [_]Uncomfortable<br>4. [_]Very uncormfortable		EF: RB<br>VN:03e07

8. Did you experience any complication during your last pregnancy?	[_] Yes<br>[_] No ↓		EF: RB<br>VN:03e08
9.What was the problem?	1. [_] Baby died<br>2. [_] Obstructed labour<br>3. [_] Post partum hemorhage<br>4. [_] Sepsis<br>5. [_] Birth canal trauma<br>6. [_] Fistula<br>7. [_] Intrapartum neonatal complication(Birth asphyxia)<br>8. [_] Other, specify ________________		EF: CB<br>VN:03e09
**Now I will ask you questions about the current pregnancy:**<br>10. Where do you plan to deliver your baby?	1. [_]Hospital<br>2. [_]TBA<br>3. [_] Home<br>4. [_] Don't know<br>5. other speciy ______________		EF: RB<br>VN: 03e10
11. Have you had any problems during this current pregnancy?	1. [_] Yes<br>0. [_] No ↓	**SKIP: If no, skip to section IV SES, EF page f q. 1**	EF: RB<br>VN: 03e11
12. What is the problem?	1. [_] Threatened abortion(bleeding)<br>2. [_] Pregnacy Induced Hypertension<br>3. [_] Malaria in pregnancy<br>4. [_] UTI in pregnancy<br>5. [_] Hyperemesis gravidarum<br>6. [_] Anaemia in pregnancy<br>7. [_] Chorioamnionitis<br>8. [_] Other, specify ________________		EF: CB<br>VN: 03e12
13. For how long was that?	ANSWER GIVEN IN:<br>____*(#(#))* (weeks)	RULE:<br>Probe till you get it as exact as possible (< 1 mo=0)	EF: RB<br>a1: Num<br>a2: Num<br>VN: 03e13
14.Did the problem result in hospitalization?	1.[_] Y es<br>0. [_] No		EF: CB<br>a3: Tx<br>a5: Tx<br>VN: 03e14

10.How long was the hospitalisation?	____*(#(#))* (days)		

Social

**QUESTION ENGLISH**	**ANSWER ENGLISH**	**RULE/ SKIP INSTRUCTION**	**COLUMN FOR CODING**
1. During the last delivery What was the transport cost to and from the health facility	………………….. INAP	**SKIP: If No, skip to q. 3**	EF: RB<br>VN: 01f01
2. What mode of transport did you use to get to the health facility?	1. [_] Private car<br>2. [_] Bus<br>3. [_] Taxi<br>4. [_] Ambulance<br>5. [_] Bicycle<br>6. [_] motorcycle<br>7. [_] On foot<br>8. [_] Other……….		EF: CB<br>VN: 01f02<br>Tick all that apply
3. What mode of transport did you use to get from the health facility to your home?	1. [_] Private car<br>2. [_] Bus<br>3. [_] Taxi<br>4. [_] Ambulance<br>5. [_] Bicycle<br>6. [_] motorcycle<br>7. [_] On foot<br>8. [_] Other……….		Tick all apply<br>EF: CB<br>VN:01f03
4. How much did it cost you to buy materials to go with to the place of delivery?	1. [_] Clothes<br>2. [_] Cotton<br>3. [_] Gauze<br>4. [_] Plastic sheet<br>5. [_] Basin<br>6. [_] Sterile surgical blade<br>7. [_] Cord clamps or ligatures<br>8. [_] Wash soap<br>8. [_] Other……….<br>…………Shs<br><br>Nothing=00		EF: CB<br>VN:01f04

5.How much did you spend during the last pregnancy?	……………..shs<br><br>I don't know =99<br>Nothing=00		EF: Num<br>VN:01f05
6.How much did it cost you to pay the health care provider?	………………Shs<br><br>Nothing=00<br><br>Paid in kind=86(specify)……….<br><br>DK=<br>Other specify	**RULE: Tick off all that apply**<br>**RULE:** Probe if alt. 1 only<br>**PROBE:**<br>Is that all?/Anything else?	EF: Num<br>VN: 01f06
7. How far was the place of birth from your home?	…………Km<br>DK=	**SKIP: If no, skip to section IV SES, EF page f q. 1**<br>**I mile =1.6km**	EF: Num<br><br>VN: 01f07
8.Did your husband escort you to the place of delivery?	1. [_] Yes<br>2. [_] No<br>3. Other specify ________		EF: CB<br>VN: 01f08
9. Who in this home decides on where the baby should be delivered from?(DO NOT PROBE TICK ALL MENTIONED)	1. [_] Paternal grandmother<br>2. [_] Maternal grandmother<br>3. [_] Husband<br>4. [_] Mother herself<br>5. [_] Others(specify)…………	RULE:<br>Write answer in months **or** years ago. Probe till you get it as exact as possible (< 1 mo=0)	EF: RB<br>a1: Num<br>a2: Num<br>VN: 01f09
**Now I will ask you questions about the current pregnancy:**<br><br>10. How far is the place of birth (you are planning to deliver from) from your home?	…………Km		EF: Num<br>VN: 01f10
11. What means of transport are you planning to use	1. [_] Private car<br>2. [_] Bus<br>3. [_] Taxi<br>4. [_] Ambulance<br>5. [_] Bicycle<br>6. [_] motorcycle<br>7. [_] On foot<br>8. [_] Other……….		EF: CB<br>VN: 01f11

SECTION: IV Socio Economic Status:
Electronic Form p f (6)

QUESTION ENGLISH	ANSWER ENGLISH	SKIP INSTRUCTION	COLUMN FOR CODING
1. How many people normally live in your housefold?	_______ (#(#))		EF: Num<br>VN: 04f01
2. How many of these are adults over 18 years?	______ (#(#))	**SKIP: If 0, skip to 4**	EF: Num<br>VN: 04f02
3. How many are children between 5 and 18 years?	_______ (#(#))	**SKIP: If 0, skip to 6**	EF: Num<br>VN: 04f03
4. How many are children less than five years old?	_______ (#(#))		EF: Num<br>VN: 04f04

English: I am now going to ask you about what you have in your household. Please answer yes if you have it and no if you do not have it. Sometimes, I'll ask you to specify how many you have of a certain subject. I am interested in the items which are in working condition.
Electronic formElectronic Form p g (7)

1. How many of the following items do you have in your household?	1. Chairs/stools ___<br>2. Foam mattresses _______<br>3. Lanterns ______	**RULE: Write correct number for all alt.s**	EF: CB<br>a1: Num<br>VN: 04g01
2. Do you have electricity in the house you are living?	1. [_] Yes<br>2. [_] No		EF: RB<br>VN: 04g02
3. Do you have any of the following in your household?	1. [_] Cupboard<br>2. [_] Bicycle<br>3. [_] Radio<br>4. [_] TV<br>5. [_] Mobile phone/ Telephone<br>6. [_] Gas heater/ electric heater<br>7. [_] Refrigerator<br>8. [_] Motorcycle/ Scooter<br>9. [_] Car/Truck	Help: Read the alternatives from the list item by item	EF: List with Y/No buttons<br>VN: 04g03

4. What is the fuel used for cooking in your household?	1. [_] Wood<br>2. [_] Charcoal<br>3. [_] Paraffin/ Kerosene<br>4. [_] Gas<br>5. [_] Electricity<br>6. [_] Other , specify<br>___________________		EF: CB<br>a6: Tx<br>VN: 04g04
12. What is the source of drinking water in your household?	1. [_] Pond, river or stream<br>2. [_] Unprotected natural spring<br>3. [_] Protected natural spring<br>4. [_] Rainwater<br>5. [_] Open or unprotected well<br>6. [_] Covered well<br>7. [_] Borehole<br>8. [_] Public tap<br>9. [_] Piped into yard / plot<br>10. [_] Piped into dwelling<br>11. [_] Bottled water<br>12. [_]Other, specify		EF: CB<br>a12: Tx<br>VN: 04g05
6. What do you do to the water before drinking it?	1. [_] Nothing<br>2. [_] Boil it<br>3. [_] Other, specify<br>___________________	**Help: Do not read out the list. Note spontaneous answer**	EF: RB<br>a3: Tx<br>VN: 04g06
7. Do you own or rent the house you live in?	1. [_] Own<br>2. [_] Rent<br>3. [_] Other, specify<br>__________________		EF: RB<br>VN: 04g07

Electronic formElectronic Form page h (3)

1. Does someone in your household own land?	1. [_] Yes<br>2. [_] No	15.	**1.** [_]<br>2. [_]		EF: RB<br>VN: 04h01
2. Do you grow crops on any land?	1. [_] Yes<br>2. [_] No	16.	**1.** [_]<br>2. [_]	**SKIP: If no, skip to q. 22**	EF: RB<br>VN: 04h02
3. Approximately how big is it?	Acres: ____________	17.		**Help: Ask for size in acres, if it is less than one write the correct 0.x**	EF: Num<br>VN:04h03

SECTION V Activities/employment:
Electronic formElectronic Form p i (9)

QUESTION ENGLISH	ANSWER ENGLISH	SKIP INSTRUCTION	COLUMN FOR CODING
1. Who is the head of the household?	1. [_] A man<br>2. [_] A woman<br>3. [_]Not applicable, specify ______________		EF: RB<br>a3: Tx<br>VN: 05i01<br>s
2. Who is the main provider of income in the household?	1. [_] Father of the child you are carrying in womb<br>2. [_] Yourself<br>3. [_] Older male relative<br>4. [_] Older female relative<br>5. [_] Other housEFold member living at home<br>6. [_] Not applic.<br>7. [_] Other, specify who<br>________________	**RULE:** Tick off her answers in the right category, do not read the list, but probe from it<br><br>**SKIP: If alternative 2, skip q. 5 and 6**	EF: RB<br>VN: 05i02
3. Is the 'main provider of income' currently employed?	1. [_] Yes<br>2. [_] No		EF: RB<br>VN05i03
4. What are the main sources of income 'the main provider' has?	1. [_] Regular employment<br>2. [_] Irregular employment<br>3. [_] Home employment<br>4. [_] Contribution from others<br>5. [_] Retirement pension/Grant<br>6. [_] Other state Grant, specify __________<br>7. [_] Relief programme<br>8. [_] No response<br>9. [_] Do not know<br>10.[_]Other, specify<br>_______________	(Alt 3 = Any income generating activity performed at home)<br><br>**RULE:**<br>Tick off her answers in the right category, do not read the list, but probe from it<br><br>Home employment=<br>Animals<br>Farming<br>Small business<br>And others work at home	EF: CB<br>a10: Tx<br><br>VN: 05i04
5. Do you earn money for yourself?	1. [_] Yes<br>2. [_] No ↓	**Not to be asked when she is the main provider q. 1 alt 2**<br>**SKIP: If no, skip to q. 7**	EF: RB<br>VN05i05

6. How do you earn money for yourself?	1. [_] Regular employment<br>2. [_] Irregular employment<br>3. [_] Home employment<br>4. [_] No response<br>5. [_] Do not know<br>6. [_] Other, specify<br>_______________	(Alt 3 = Any income generating activity performed at home)<br><br>**RULE: Tick off all that apply**	EF: CB<br>a10: Tx<br><br>VN: 05i06
7. Does your household have any other sources of income?	1. [_] Yes<br>2. [_] No ↓	**SKIP: If no, skip to 9**	EF: RB<br>VN05i07
8. What kind of sources is that?	1. [_] Regular employment<br>2. [_] Irregular employment<br>3. [_] Home employment<br>4. [_] Contribution from others<br>5. [_] Bussiness<br>6. [_] Retirement pension/Grant<br>7. [_] Other state Grant, specify __________<br>7. [_] Relief programme<br>8. [_] Business<br>9. [_] No response<br>10. [_] Do not know<br>11.[_]Other, specify<br>_______________		EF: CB<br>a10: Tx<br><br>VN: 05i08
9. Do you work on land?	1. [_] Yes<br>2. [_] No ↓	**SKIP: If no, skip to 11**<br>**ADRESSED TO THE MOTHER**	EF: RB<br>VN05i09
10. Is the land you work on your own land or rented land?	1. [_] Own land<br>2. [_] Rented land<br>3. [_]Other, specify<br>_______________	**ADRESSED TO THE MOTHER**<br>**Your own land refers to the hh's land.**	EF: RB<br>a4: Tx<br>VN: 05i10
11. Do you work in a small or large business?	1. [_] Small<br>2. [_] Large<br>3. [_] Do not work in a business	**SKIP: If alt. 3 ticked off, skip to 13**<br>**ADRESSED TO THE MOTHER**	EF: RB<br>VN05i011
12. Do you work in your own business, family business or someone else's business?	1. [_] Own business<br>2. [_] Family business<br>3. [_] Someone else's business<br>4. [_] Does not apply	**ADRESSED TO THE MOTHER**	EF: CB<br>a4: tx<br><br>VN:05i12

	5.[_]Other, specify<br>_______________		
13. What is your monthly salary?	___________USH<br><br>(Each site specify their own currency)	**Remember to make skip instruction at Country above**<br><br>**ADRESSED TO THE MOTHER**	EF: RB<br>a1-a4: Num<br><br>VN: 05i13U<br>Site specific currency with Site specific Variable names 05i13S/Z/B
14. How much of your monthly earnings are you spending on yourself only?	___________USH<br><br>(Each site specify their own currency)	**ADRESSED TO THE MOTHER**	EF: RB<br>a1-a4: Num<br><br>VN: 11i14U<br>05i14B<br>05i14Z<br>11i14S
15. Do you usually work throughout the year, or do you work seasonally, or only once in a while or does it not apply to you?	1. [_] Throughout the year<br>2. [_] Seasonally<br>3. [_] Once in a while<br>4. [_] Does not apply<br>5. [_]Other, specify<br>_______________		EF: RB<br>a5: Tx<br><br>VN: 01i15<br><br>Specify: 05i15a5s
16. How much does your household spend a normal month on the following expenses?	1. [_] Feeding __<br>2. [_] Housing __<br>3. [_] Schooling __<br>4. [_] Clothing __<br>5. [_] Water and Drainage ____<br>6. [_] Electricity _______<br>7. Rent of land ___<br>8. Modern medicine _______<br>9. Traditional medicine _______<br>10. [_] Social acitivites ___<br>11. [_] Other, specify	Alt 1: Write the estimated amount from 0 and upward<br><br>Rule: Do not ask about electricity to hh without electricity<br><br>Probe for baptisms, weddings, funerals/burials and specify that under social, specify. Also ask for other expenses	EF: CB<br>a1-a8: Tx<br>(allows for DNK = xx for alternatives)<br><br>VN: 05i16<br><br>Example Uganda:<br>a1: 05i16a1Ug

SECTION VI Questions on use of clinical/medical services

Electronic formElectronic Form p. j (10)

RULE for section: If she refuses to answer; tick in "Missing" under the question mark alternative

QUESTION ENGLISH	ANSWER ENGLISH	SKIP INSTRUCTION	COLUMN FOR CODING
1. Have you attended any sessions at the antenatal care clinic (ANC)?	1. [_] Yes<br>2. [_] No ↓	**SKIP: If no skip to q. 3**	EF: RB<br>VN: 06j01
2. How many times have you been there in this pregnancy?	__________(#)		EF: Num<br>VN: 06j02
3. Do you use a treated \bed net for yourself?	1. [_] Yes<br>2. [_] No<br>3. [_] Sometimes		EF: RB<br>VN: 06j03

SECTION VII Previous child mortality

SKIP: If section 2 q.1=No previous children, skip this section and jump to SECTION VIII **unscrupulous**

Electronic formElectronic Form p. k (11)

QUESTION ENGLISH	ANSWER ENGLISH	SKIP INSTRUCTION	COLUMN FOR CODING
1. Have any of your children who were born alive died?	1. [_] Yes<br>2. [_] No ↓	**SKIP: If no, skip to SVIII; EF page l**	EF: RB<br>VN: 07k01
2. How many of your children have died?	1. [_] 1 child has died<br>2. [_] More than 1 child has died, specify number	**If alternative 1 given, disable q. 5**	EF: Num<br>VN: 01k02
3. May I ask how old your lastborn: (*if more than 1 child death*) child was when he/she died? **R**	ANSWER GIVEN IN:<br>1. _____ *(#(#))* days<br>OR:<br>2. _____ *(#(#))* wks<br>OR:<br>3. _____ *(#(#))* mo<br>OR:	**If she does not remember:**<br>**PROBE and fill in right cat below**<br><br>**Rule: Lastborn refers to the one "before the one**	EF: CB<br>a1: Num<br>a2: Num<br>a3: Num<br>a4: Num<br>VN: 01k03

	4. _____ *(#(#))*years<br>5. [_] Do not know	**she is carrying"/gave birth to ≤ 1wk ago who might have died**	
**4. PROBE:**<br>Was he/she less than one month, between one month and one year or between one year and 5 years:	1. [_] Less than one month<br>2. [_] Greater than or equal to one month and less than one year<br>3. [_] Greater than or equal to one year and less than five years<br>4. [_] Greater than or equal to five years	**Disable this one if q. 3 answered**	EF: RB<br>VN: 01k04
**5. IF > 1 child deaths**<br>**Note right age category for the other children from alt 1-4 above**	1. Alt: ____Child 2<br>2. Alt: ____Child 3<br>3. Alt: ____Child 4<br>4. [_] ≥ 5 child deaths<br>Comment:<br>________________	Age categories used:<br>1. [_] Less than one month<br>2. [_] Greater than or equal to one month and less than one year<br>3. [_] Greater than or equal to one year and less than five years<br>4. [_] Greater than or equal to five years	EF: CB<br>a1: Num<br>a2: Num<br>a3: Num<br>a4: Tx<br><br>VN: 01k05
**6. What was the main sickness or reason which led to death for the child(ren) you have lost?**	1. [_] Child 1 _____<br>2. [_] Child 2 _____<br>3. [_] Child 3 _____<br>4. [_] Child 4 _____<br>5. [_] Child 5 _____<br>6. [_] More than 5, comment _____		EF: CB<br>Alternatives: tx<br>VN: 01k06

Electronic formElectronic Form p l (12)
VIII Observation: Help text: "Thank you, now I am going to ask you some questions about your house and it's surroundings."

1. QUESTION ENGLISH	2. ANSWER ENGLISH	5. SKIP INSTRUCTION	6. COLUMN FOR CODING

If a single room hut, do not ask, just note down::<br>1. How many rooms do you have in your housEFold which are used for sleeping?	_______ *(#(#))*		EF: Num<br>VN: 01l01
2. Do you have a toilet?	1. [_] Yes<br>2. [_] No ↓	**SKIP: If no, skip to q. 5**	EF: RB<br>VN: 01l02
3. May I please see your toilet/latrine? (U)<br>What type of toilet is it? (BF/Z)	1. [_] Nothing<br>2. [_] Open pit<br>3. [_] Pit latrines<br>4. [_] VIP latrine<br>5. [_] Flush toilette<br>6. [_] Other, specify<br>________________		EF: RB<br>VN: 01l03
4. Do you share this/your toilet with any neighbouring housEFolds?	1. [_] Yes<br>2. [_] No		EF: RB<br>VN: 01l04
5.If yes,approximately how many people use this toilet?	……….people		EF:RB<br>VN:01l05
6. Where do you wash your hands? *(If within reach/existing):* May I please see it?	1. [_] Not within reach<br>2. [_] Insufficient water supply, no soap<br>3. [_] Sufficient water supply, no soap<br>4. [_] Sufficient water supply, soap	Tick off for the type of washing equipment or lack of washing equipment you see<br><br>Not within reach=she normally never wash hands because of the distance after a visit to the toilet	EF: RB<br>VN: 01l06

SECTION VIII CARE DURING DELIVERY

Electronic formElectronic Form p. e (5)

**QUESTION ENGLISH**	**ANSWER ENGLISH**	**RULE/ SKIP INSTRUCTION**	**COLUMN FOR CODING**

Where did you deliver from?	1. [_] Hospital<br>2. [_] Health center<br>3. [_] TBA<br>4. [_] Private clinic<br>5. [_] Home<br>6. [_] On route to hospital or health center<br>7. [_] Other(specify)<br>……………………	**SKIP: If No, skip to q. 3**	EF: RB<br>VN: 08e01
Who assisted you during delivery?	1. [_] Health professional<br>2. [_] TBA<br>3. [_] Relatives<br>4. [_] Other(specify)<br>…………………..		EF: Num<br>VN: 01e02
At what time did labour begin?	[_] Day<br>[_] Mid day<br>[_] Evening<br>[_] Night		EF: RB<br>VN: 08e03
After how long did delivery take place after onset of labour?	[_] Hours<br>[_] Days<br>[_] I don't know<br>[_] Other specify……………		EF: RB<br>VN: 08e04
Where you blood transfused during/after birth?	[_] Yes<br>[_] No		EF: RB<br>VN: 08e05
At what time was the baby born?	[_] Day<br>[_] Mid day<br>[_] Evening<br>[_] Night		EF: RB<br>VN: 08e06
Did you(the mother) experience any of the following signs during the first month after delivery?	[_] Abnormal vaginal bleeding<br>[_] loss of consciousness<br>[_] Fever<br>[_] Swelling<br>Other specify<br>Convulsions		EF: RB<br>VN: 08e07
On which surface did the delivery take place?	[_] Labour bed<br>[_] Labour bed with plastic sheet/cloth/makintosh<br>[_] Plastic sheet/cloth/makintosh on floor<br>[_] Bare floor<br>[_] Bare floor<br>[_]Other (specify)………		EF: RB<br>VN: 08e08

What was used to cut the cord?	1. [_] Un used new razor blade<br>2. [_] Used razor blade<br>3. [_] Scissors<br>4. [_] Knife<br>5. [_] Other (specify)…………<br>6. [_] Don't know		EF: RB<br>VN: 08e09
What was used to tie the cord?	1. [_] Cloth strip<br>2. [_] Thread<br>3. [_] cord ligature<br>Other(specify)…………<br>4. [_] Don't know(don't remember)		EF: RB<br>VN: 08e10
11.What was put on the cord to help it heal?	1. [_] Nothing<br>2. [_] Medical drugs<br>3. [_] Powder<br>4. [_] Ash<br>5. [_] Salty water<br>6. [_] Spirit<br>cowdung<br>Other(specify)………………		EF: RB<br>VN: 08e11
9. Did the attendant at delivery counsel you on the following?	1. [_] Breast feeding<br>2. [_] Keeping the baby warm<br>3. [_] Cord care<br>4. [_] Danger signs of the newborn<br>5. [_] Importance of health facility visit<br>6. [_] family planning<br>7. [_] Immunisation<br>8. [_] Vitamin A supplementation<br>Other specify		EF: RB<br>VN: 08e12

SECTION IX New borne care practices

SKIP instruction: If section II, question 1 = Alternative 2 Skip to question 5

English:

Now I am going to ask you questions about the last child you had

Langi

Baseline survey

Electronic formElectronic Form p. e (5)

1. QUESTION ENGLISH	2. ANSWER ENGLISH	5. RULE/ SKIP INSTRUCTION	6. COLUMN FOR CODING
1. Is your baby alive?	1. [_] Yes<br>2. [_] No		EF: RB<br>VN: 09e01
2. How old is your babybaby in months?	_______		EF: Num<br>VN: 09e02

3. What is the sex of your baby?	ANSWER GIVEN IN:<br>1. [_] Male<br>2. [_] Female		EF: RB<br>VN: 09e03
4. What was the mode of delivery?	1. [_] Normal delivery<br>2. [_] Caesarian<br>3. [_] Assisted vaginal delivery		EF: RB<br>VN: 09e04
5.How many pregnancies have you had?	………………		EF: CB<br>VN: 09e05
6. How many children have you had?	………………….		EF: RB<br>VN: 09e06
7. How many live children have you had?	________________		EF: Num<br>VN: 09e07
8. Was the baby weighed after immediately delivery?	1. [_] Yes<br>2. [_] No		EF: RB<br>VN: 09e08
8b.If yes what was the birth weight?	……………(kgs)		
8c. What were you told that weight meant?	[_] Low birth weight<br>[_] Normal weight<br>[_] Large for gestation age<br>[_] Not told		
9. Did the baby have any problem with breathing or crying immediately after delivery?	1. [_] Yes<br>2. [_] No<br>3. [_] Don't know		EF: RB<br>a3: Tx<br>a5: Tx<br>VN: 09e09
10. Did anyone do the following things on the baby after delivery?	1. [_] Cleaning<br>2. [_] Blow air in the mouth<br>3. [_] Try to bring up the baby's life<br>Other specify		EF: CB<br>VN: 09e10
11. Where was the baby placed immediately after delivery?	1. [_] On the ground<br>2. [_] On the stomach/chest of the mother<br>3. [_] At the side of her mother<br>4. [_] Someone else<br>Other specify)…………….		EF: RB<br>VN: 09e11
11b.Was the baby dried immediately after delivery?	[_] yes<br>[_]No<br>Don't know		EF: RB<br>VN: 09e11

12.Was the baby wrapped in a cloth after delivery before the placenta came out?	1. [_] Yes<br>2. [_] No<br>3. [_] don't know		EF: RB<br>VN: 09e12
13. What was used to tie the cord?	1. [_] Piece of thread<br>2. [_] Piece of cloth<br>3. [ ] Piece of glove<br>4. [_] Clamps/ ligatures<br>5. [_] Other specify…………………		EF: RB<br>VN: 09e13
14. What was used to cut the cord?	1. [_] New razor blade<br>2. [_] Old razor blade<br>3. [_] Pair of scissors<br>4. [_] Spear grass<br>5. [_] Knife<br>4. [_] others……………..		EF: RB<br>VN: 09e14
15. Was the option used for tying the cord already sterilized	1. [_] Yes<br>2. [_] No<br>3. [_] don't know		EF: RB<br>VN: 09e15
15b. If yes, how was it sterilized?	1. [_] Boiled<br>2. [_] Deeped in spirit<br>3. [ ] Prepacked sterile material<br>4. [_] Other specify………….		
16. Was anything applied on the cord just after cutting and tying it?	1. [_] yes<br>2. [_] No		EF: RB<br>VN: 09e16
16b. If yes by whom?	[_] Mother of baby<br>[_] Father of baby<br>[_] Mother in law<br>[_] Sister<br>[_] grandmother<br>[_] Friend<br>[_]TBA<br>[_]Health worker<br>[_] Other(specify)		
17. What was applied to the cord just after cutting it?	1. [_] Ghee<br>2. [_] Ash<br>3. [_] Medicine to smear<br>4. [_] Dung<br>5. [_] cooking oil<br>6. [_] Salty water<br>7. [_] surgical spirit<br>8. [_] Chlorhexidine<br>9. [_] Others		EF: RB<br>VN: 09e17

	specify…………………		
17b.Was anything applied to the skin of the baby?	[_]Yes<br>[_]No		
17c.What was applied to the cord/skin just after cutting it?	1. [_] Ghee<br>2. [_] Ash<br>3. [_] Medicine to smear<br>4. [_] Dung<br>5. [_] cooking oil<br>6. [_] Salty water<br>7. [_] surgical spirit<br>8. [_] Chlorhexidine<br>9. [_] Others<br>specify…………………		
18. How long did you take to bath the baby after delivery?	1. [_] In the first 6 hours<br>2. [_] Between 6-24 hours<br>3. [_] Greater than 24 hours		EF: RB<br>VN: 09e18
18b. Who bathed the baby?	[_] Mother of baby<br>[_] Father of baby<br>[_] Mother in law<br>[_] Sister<br>[_] grandmother<br>[_] Friend<br>7.[_] Other(specify)		
19. How was warmth maintained after birth?	1. [_] Direct Contact with mother's skin<br>2. [_] Wrapping in cloth<br>3. Electric warmer<br>3. [_] Other(specify)………..		EF: RB<br>VN: 09e19
19b.Was head covering done all the time for the baby?	[_]Yes<br>[_]No<br>[_]Don't know	**Head covering includes both capes & cloth/sheet**	
19c.Who was the person responsible for that?	[_] Mother of baby<br>[_] Father of baby<br>[_] Mother in law<br>[_] Sister<br>[_] grandmother<br>[_] Friend<br>Health professional<br>7.[_] Other(specify)		
20. who did the baby sleep with at night in the first week of life?	1. [_] Slept with the mother<br>2. [_] Slept alone<br>3. [_] the baby with a different person		EF: RB<br>VN: 09e20

	4. Other specify		
21. How long after delivery did you put the baby on breast	1. [_] During the first hour<br>2. [_] After an hour but on the same day<br>3. [_] After one day<br>4. [ ] Never		EF: RB<br>VN: 09e21
22.Who was responsible for that descision?	[_] Mother of baby<br>[_] Father of baby<br>[_] Mother in law<br>[_] Sister<br>[_] grandmother<br>[_] Friend<br>Health professional<br>7.[_] Other(specify)		EF: RB<br>VN: 09e22
23. Did you express the first breast milk and discard?	1. [_] Yes<br>2. [_] No<br>3. Other specify<br>______________		EF: RB<br>VN: 09e23
24. Did you give the baby anything else in the first month apart from breast milk to feed the baby?	1. [_] Yes<br>2. [_] No<br>3.[ ] Don't know		EF: RB<br>VN: 09e24
24b.What was given apart from breast milk to feed the baby?	[_]Milk(other than breastmilk)<br>[_] Plain water<br>[_]Sugar and salt solution<br>[_]Gripe water<br>[_]Sugar or glucose water<br>[_]Fruit juice<br>[_]Infant formular<br>[_]Tea<br>[_]Honey<br>[_]Others(specify)…………		EF: CB<br>VN: 09e24
25. On the first day after births, did did any health provider examine you (the mother)?	1. [_] Yes<br>2. [_] No		EF: RB<br>VN: 09e25
26. Did any health worker visit you to provide care or advice in the first month after delivery?	1. [_] Yes, health worker<br>2. [_] Yes, village health team /CHW<br>1. [_] Yes, TBA<br>3. [_] No<br>4. Don't know<br>Other specify		EF: CB<br>VN: 09e26
27.Within the 1st month after	1. [_] Yes		EF: RB

delivery, did you ever take your child to a health facility for check up?	2. [_] No		VN: 09e27
28. How old in days was the baby at the time of the first check at a healthy facility or home?	…………………………. (weeks)		EF: Num<br>VN: 09e28
29.How many times did your baby have a check up at the health facility or home in the first month o life?	………………………..		EF: Num<br>VN: 09e29
30.During the baby's check, was the following done?	1. [_]Vitamin A<br>2. [_] Immunisation for the baby?		EF: CB<br>VN: 09e30
31.Did you ever feed the baby with a bottle during the first one month of the baby's life?	1. [_] Yes<br>2. [_] No<br>3. [ ] Don't know		EF: RB<br>VN: 09e31
32.During the first pregnancy, where you ever advised on how to breastfeed the baby?	1. [_]Yes<br>2. [_]No<br>3. Don't know		EF: RB<br>VN: 09e32
33.Do people who regularly care for the baby wash their hands before handling the baby?	1. [_] Yes, all the time<br>2. [_] Yes, Sometimes<br>3. [ ] No<br>4. [ ] Don't know		EF: RB<br>VN: 09e33
34.What do they use to wash their hands?	1. [_] Water only<br>2. [_] Water and soap<br>3. [ ] Sanitizer<br>3. [_] Other(specify)…………..		EF: RB<br>VN: 09e34
35.New born babies often get ill and some illnesses are serious to their lives in the first month of life.Which signs may show that a newborn's health may be in danger?	1. [_]Convulsions or fits<br>2. [_]Inability to feed adequately or cessation of suckling<br>3. [_]Lethargy(very weak) loss of consciousness<br>4. [_]Redness of the umbilicus extending to the skin of the abdomen<br>5. [_]Persistent vomiting abdominal distention<br>6. [_]Difficulty breathing<br>7. [_]Fever<br>unusually cold body temperature<br>8.[_]Other(specify)………	**DO NOT PROBE. CIRCLE ALL MENTION ED**	EF: CB<br>VN: 09e35

36.Did your child have any of the following signs in the first month of life?	1. [_]Convulsions or fits<br>2. [_]Inability to feed adequately or cessation of suckling<br>3. [_]Lethargy(very weak) loss of consciousness<br>4. [_]Redness of the umbilicus extending to the skin of the abdomen<br>5. [_]Persistent vomiting abdominal distention<br>6. [_]Difficulty breathing<br>7. [_]Fever<br>unusually cold body temperature<br>8.<br>[_]Other(specify)…………….…		EF: CB<br>VN: 09e36
**Diarrhoea**			
1.During the last month did <NAME> have diarrhoea?(more than three watery stools, not typically breastmilk stools, in <24hours)	1. [_] Yes<br>2. [_]No ↓		EF: RB<br>VN: 10f01
Did <NAME> pass any watery stools?	[_] Yes<br>[_] No		EF: RB<br>VN: 10f02
The day <NAME> had most loose or watery stools, how many loose or watery stools did <NAME> pass?	……………(##)		EF: Num<br>VN: 10f03
Did any of the stools contain blood?	[_] Yes<br>[_] No		EF: RB<br>VN: 10f04
Were the stools of different consistency than before <NAME> fell ill with diarrhoea?	[_] Yes<br>[_] No		EF: RB<br>VN: 10f05
Did the illness interfere with <NAME>'s ability to drink or eat?	[_] Yes<br>[_] No		EF: RB<br>VN: 10f06
Did you seek treatment for < Name>	[_] Yes<br>[_] No↓		EF: RB<br>VN: 10f07
Where did you go?	[_] Relatives and friends<br>[_] Traditional healer<br>[_] Drugshop/Pharmacy<br>[_] Government or private clinic/surgery/community		EF: CB<br>VN: 10f08

	health centre including general practitioner<br>[_] The emergency/outpatient department of a hospital<br>[_] Other, specify…………		
Was the child admitted to a hospital?	[_] Yes<br>[_] No ↓		EF: RB<br>VN: 10f09
Please give the name of the hospital?	[_]<br>[_]<br>[_]<br>[_]<br>[_]<br>[_]<br>[_]<br>[_]		EF: RB<br>VN: 10f10
Was this the nearest health unit?	[_] Yes<br>[_] No		EF: RB<br>VN: 10f11
Why did you go there?	[_] Health services better than at the nearest health unit<br>[_] Transport was available<br>[_] The nearest health unit is more expensive than the other I went to<br>[_] I wanted to go to the biggest hospital I can afford<br>[_] I do not trust the people at the nearest health unit<br>[_] Other, specify		EF: CB<br>VN: 10f12
ALT1:How many days did the diarrhoea last/<br>ALT 2: How many days has the diarrhoea lasted?	[_]Finished: Duration in days……………<br>[_] Not finished: Duration in days:…………….		EF: Num<br>VN: 10f13
Is your child still alive?	[_] yes<br>[_] No↓		EF: RB<br>VN: 10f14
Has your child changed in any way after that illness?	[_] yes<br>[_] No ↓		EF: RB<br>VN: 10f15
In which way?	[_] stopped breast feeding<br>[_] Became smaller<br>[_] Other specify…………….		EF: RB<br>VN: 10f16
Who in this home decides on where the baby should be treated from?	[_] Paternal grandmother<br>[_] Maternal grandmother<br>[_] Husband<br>[_] The mother her self<br>[_] thers(specify)…………..		EF: RB<br>VN: 10f17

1. During the last two weeks that ended yesterday morning, did <NAME> have cough?	1. [_] Yes<br>2. [_] No		EF: RB<br>VN: 10g01
2. During the last two weeks that ended yesterday morning, did <NAME> have fast or difficult breathing?	1. [_] Yes<br>2. [_] No ↓		EF: RB<br>VN: 10g02
3. Did the illness interfere with <NAME>'s ability to drink or eat?	1. [_] Yes<br>2. [_] No		EF: RB<br>VN: 10g03
4. Was <NAME> admitted to a hospital for the illness?	1. [_] Yes<br>2. [_] No ↓		EF: RB<br>VN: 10g04
5. Please give name of hospital?	1. [_] Lira hospital<br>2. [_] _________________		EF: RB<br>VN: 10g05
6. Was this the nearest health unit?	1. [_] Yes<br>2. [_] No ↓		EF: RB<br>VN: 10g06
7. Why did you go there?	1. [_] Health cervices better than at the nearest health unit<br>2. [_] Transport was available<br>3. [_] The nearest health unit is more expensive than the one I went to<br>4. [_] I wanted to go to the biggest hospital I can afford<br>5. [_] I do not trust the people at the nearest health unit<br>6. [_] Other, specify		EF: RB<br>VN: 10g07
8. During this period of illness you have described, did you change the way you were feeding your child in any way?	1. [_] Yes<br>2. [_] No ↓		EF: RB<br>VN: 10g08
9. In which way?	1. [_] Stopped breast feeding<br>2. [_] Stopped non-human milk<br>3. [_] Stopped other liquids<br>4. [_] Stopped solid foods<br>5. [_] Only breast fed at night<br>6. [_] Began giving other liquids<br>7. [_] Began giving solid foods		EF: CB<br>VN: 10g09

	8. [_] Other, specify		
10. During the period of illness did you feed your baby more often, more seldom than or just as often as before the illeness started?	1. [_] More often<br>2. [_] More seldom than before the illness started<br>3. [_] Did not change feeding frequency.		EF: RB<br>VN: 10g10
1.Did the child experience any vomiting(more than three episodes of forceful vomiting in <2hrs)?	[_] Yes<br>[_] No		EF: RB<br>VN: 10h01
2.Did the child experience any abnormal bEFaviour like excess irritability or abnormal sleepiness?	[_]Yes<br>[_]No		EF: RB<br>VN: 10h02
3.Did the child experience feeding difficulty i.e unable to feed as well as usual or expected	[_]Yes<br>[_]No		EF: RB<br>VN: 10h03
4.Did this child experience any convulsion?	[_]Yes<br>[_]No		EF: RB<br>VN: 10h04
5.Did this child have any yellowing of eyes?	[_] Yes<br>[_] No		EF: RB<br>VN: 10h05
6.Did the child experience any swelling or pus discharge from the eyes?	1.[_] Yes<br>2. [_]No		EF: RB<br>VN: 10h06
7.Did the child experience any fast breathing?	[_]Yes<br>[_]No		EF: RB<br>VN: 10h07
8.Did the child experience any noisy breathing?	[_]Yes<br>[_]No		EF: RB<br>VN: 10h08
9.Did the child experience any chest indrawing?	[_]Yes<br>[_]No		EF: RB<br>VN: 10h09
10.Did the child's gums or tongue turn dark blue in colour?	[_]Yes<br>[_]No		EF: RB<br>VN: 10h10
11.Did the child experience any difficulty in arousal on stimulation?	[_]Yes<br>[_]No		EF: RB<br>VN: 10h11
12.Did the child experience any inability to attach or suck adequately at breast?	[_]Yes<br>[_]No		EF: RB<br>VN: 10h12
13.Did the child ever become uncounscious?	[_]Yes<br>[_]No		EF: RB<br>VN: 10h13
14.Did the child experience any pus over the umbilical area?	[_]Yes<br>[_]No		EF: RB<br>VN: 10h14
15.Did the child ever become rigid?	[_]Yes<br>[_]No		EF: RB<br>VN: 10h15

16.Did the child ever become febrile?	[_]Yes<br>[_]No		EF: RB<br>VN: 10h16
17.Did the child ever become unusually cold?	[_]Yes<br>[_]No		EF: RB<br>VN: 10h17
18.Did you seek treatment for the childs illness?	[_]Yes<br>[_]No		EF: RB<br>VN: 10h18
19.Where did you go?	[_]Govt hospital<br>[_]Health center<br>[_]Other public (specify)<br>[_]Private hospital/clinic<br>[_]Drug shop<br>[_]Other/traditional healer home		EF: RB<br>VN: 10h19
20.If more than one, in what order?	……………………		EF: RB<br>VN: 10h20

SECTION XI: Hospitalizations

Electronic formElectronic Form p. J (5)

QUESTION ENGLISH	ANSWER ENGLISH	SKIP INSTRUCTION	COLUMN FOR CODING
1. Since birth has <NAME> ever been admitted to hospital?	1. [_] Yes<br>2. [_] No ↓	**SKIP: If no, skip to S V**	EF: RB<br>VN: 11J01
2. How many times has <NAME> been admitted to hospital?	_____ (#(#))		EF: Num<br>VN: 11J02
3. How old in weeks was your baby (each time) when he/she was in hospital?	1. [_] 1st time _______<br>2. [_] 2nd time _______<br>3. [_] 3rd time _____<br>4. [_] 4th time _______<br>5. [_] 5th time _______<br>6. [_] 6th time _______<br>7. [_] 7th time _______<br>8. [_] 8th time _______	**RULE: Tick off all that apply**	EF: CB<br>a1: Num<br>a2: Num<br>a3: Num etc.<br><br>VN: 11J03

4. For how many days was <NAME> (each time) in hospital?	1. [_] 1st time _______<br>2. [_] 2nd time _______<br>3. [_] 3rd time _____<br>4. [_] 4th time _______<br>5. [_] 5th time _______<br>6. [_] 6th time _______<br>7. [_] 7th time _______<br>8. [_] 8th time _______		EF: CB<br>a1: Num<br>a2: Num<br>a3: Num etc.<br><br>VN: 11J04
5. What was the reason <NAME> was in the hospital each time:		**RULE: ENTER THE CORRECT NUMBER FROM THE LIST BELOW**<br>1 = Diarrhoea<br>2 = Pneumonia/ "Cought and difficult breathing"<br>3 = Malaria<br>4 = Accident<br>5 = Specify what ____________	EF: CB<br>a1: Tx<br>a2: Tx<br>a3: Tx etc.<br>Allows for DNK for alternatives<br><br>VN: 11J05

Baby's born weighing less than 2.5kg at birth

Electronic formElectronic Form p. k (13)

QUESTION ENGLISH	ANSWER ENGLISH	SKIP INSTRUCTION	COLUMN FOR CODING
1. Was your baby born at term?	1. [_] Yes<br>2. [_] No ↓<br>96. [_] Don't know	**SKIP: If no, skip to S V**	EF: RB<br>VN: 12k01
2. How many weeks before the expected date was(name)born?	______ (#(#))		EF: Num<br>VN: 12k02
3. Did you make extra visits to the health facility because your baby was small?	1. [_]Yes<br>2. [_]No	**RULE: Tick off all that apply**	EF: RB<br>.<br><br>VN: 12k03

4. Were you given any information on how to care for small babies?	1. [_] 1Yes<br>2. [_] 2No<br>Don't know		EF: CB<br>a1: Num<br>a2: Num<br>a3: Num etc.<br><br>VN: 12k04
5. What were you told?	1. [_] more frequent breastfeeding<br>2. [_] skin to skin care<br>3. [_] delay first birth for a week or longer<br>4. [_] take baby to health facility<br>5. [_] Extra PNC visits<br>6. [_] Other(specify)…….<br>7. [_]Don't remember?<br>7. [_] 7th time _______<br>8. [_] 8th time _______	____________	EF: CB<br>a1: Tx<br>a2: Tx<br>a3: Tx etc.<br>Allows for DNK for alternatives<br><br>VN: 12k05<br>**Tick off all that apply**
6.Do you feel the information you received was useful to help you care for the baby well?	1. [_]Yes, very useful<br>2. [ ]Yes, somewhat useful<br>2. [_]No, not useful		EF: RB<br>VN: 12k06
7.Were you able to do all that you were told?	1. [_]Yes<br>2. [_]No		EF: RB<br>VN: 12k07
8.If no why?	Think of some viable options and then add other specify		EF: RB<br>VN: 12k08
9. EFe was your baby taken to a health facility for special care?	[_]Yes<br>[_]No		EF: RB<br>VN: 12k09
10.Did (Name) receive Kangaroo Mother Care(KMC) at a health facility?	1. [_]Yes<br>2. [_]No		EF: RB<br>VN: 12k10
11. When did KMC start?	……(hours)		EF: Num<br>VN: 12k11
12.For how longdid(Name) receive KMC in the health facility?	…….. (days)		EF: Num<br>VN: 12k12

13.How long did KMC continue at home?	…….(days)		EF: Num<br>VN: 12k13

SECTION XIII: Mama Kits-Availability and knowledge in the community

Electronic formElectronic Form p. l (13)

Do you know about mama kits?	1. [_] Yes<br>2. [_] No		EF:RB<br>VN:13l01
If yes, did you use it during your previous delivery?	1. [_] Cotton wool<br>2. [_] Gauze<br>3. [_] Sterile gloves<br>4. [_] Cord ligature<br>5. [_] Plastic sheet<br>6. [_] Surgical blades<br>7. [_] Washing soap<br>7. [_] Tetracycline<br>8. [_] Sanitary pads		EF:CB<br>VN:13l02
Do you have one here with you?	1. [_] Yes<br>2. [_] No		EF:RB<br>VN:13l03
Where did you get it?	1. [_] VHT<br>2. [_] Drug shop/ pharmacy<br>3. [_] Health center during anatenatal<br>4. [_] Health center during birth<br>5. [_] Other, specify………….		EF:RB<br>VN:13l04
What do the health workers use for hand washing in the labor room?	1. [_] Soap and water<br>2. [_] Water only<br>3. [_] Others, specify……………..		EF:RB<br>VN:13m01
Are there disinfectants/antiseptics available for infection control in the labor room	1. [_] Jik<br>2. [_] 70% alcohol<br>3. [_] Iodine<br>4. [_] Surgical spirit<br>2. [_]<br>3. [_] Others, specify……………..		EF:CB<br>VN:13m02

How often do the HWs disinfect their working surfaces?	1. [_] Daily<br>2. [_] Sometimes<br>3. [_] Often<br>4. [ ] After every delivery<br>4. [_] Others, specify……………..		EF:RB<br>VN:13m03
Availability of protective gear	1. [_] Aprons<br>2. [_] Scrubs<br>3. [_] Clinical coat<br>4. [_] Gloves<br>5. [_] Plastic sheet<br>6. [_] Others, specify……………..	**Rule: Do not read out, just observe**	EF:RB<br>VN:13m04
How often do you clean/disinfect the labor bed	1. [_] Daily<br>2. [_] Reguraly<br>3. [_] Every after a delivery<br>4. [_] Others, specify……………..		EF:RB<br>VN:13m05
How often do you clean/disinfect labor room syringe bulb	1. [_] Daily<br>2. [_] Reguraly<br>3. [_] Every after a delivery<br>4. [_] Others, specify……………..		EF:RB<br>VN:13m06

RECORD OBSERVATION: RULE: Do not read out loud!

Electronic formElectronic Form p m (13)

QUESTION ENGLISH	ANSWER ENGLISH	SKIP INSTRUCTION	COLUMN FOR CODING
6. Main material of the floor:	1. [_] Earth/Dung<br>2. [_] Cement<br>3. [_] Tiles<br>4. [_] Rudimentary wooden<br>5. [_] Finished wooden<br>6. [_] Carpet/Vinyl<br>7. [_] Other, specify	**Tick off 1 alternative only**<br><br>**(Choose alternative which makes up > half of the floor)**	EF: RB<br>a7: Tx<br><br>VN: 01m06
7. Main material of the roof:	1. [_] Thatch, grass<br>2. [_] Iron sheets<br>3. [_] Tiles<br>4. [_] Concrete<br>5. [_] Wood<br>6. [_] Other, specify	**Tick off 1 alternative only (Choose alternative which makes up > half of the roof)**	EF: RB<br>A6: Tx<br><br>VN: 01m07
8. Main material of the walls:	1. [_] Mud and pole<br>2. [_] Wood<br>3. [_] Tin<br>4. [_] Bricks without mortar	**Tick off 1 alternative only (Choose alternative which**	EF: RB<br>a7: Tx<br><br>VN: 01m08

	5. [_] Burnt brick with mortar<br>6. [_] Plastered walls<br>7. [_] Other, specify	**makes up > half of the walls)**	
9. Status of toilet:	1. [_] Visible faeces<br>2. [_] Not visible faeces	**Tick off 1 alternative only**	EF: RB<br>VN: 01m09
10. Status of compound:	1. [_] Littered<br>2. [_] Not littered<br>3. [_] Animal faeces on the ground<br>4. [_] Human faeces on the ground	**Tick off all that apply**	EF: CB<br>VN: 01m10
11. Main material of windows:	1. [_] No material<br>2. [_] Wood<br>3. [_] Glass<br>4. [_] Other, specify		EF: RB<br>VN: 01m11
12. Main material of doors:	1. [_] No doors<br>2. [_] Only outer doors<br>3. [_] Outer and inner doors<br>4. [_] Other, specify		EF: RB<br>VN: 01m12
13. The data collector ticks off the type of house the mother lives in:	1. [_] Shack<br>2. [_] Traditional Hut<br>3. [_] Semi-permanent house<br>4. [_] Permanent house<br>5. [_] Other, specify	**Tick off 1 alternative only**	EF: RB<br>VN: 01m13
14. Other comments:	_______________		EF: Tx<br>VN: 01m 14

READ OUT LOUD:
Thank you very much for your help! This is a great help for us!
Be free to thank/greet in local language to round off nicely!

A: Survival PLUSS   Post Recruitment Questionnaire
List of abbreviations
EF = Electronic form
Radio Button (RB), One alternative only
Check Boxes (CB), Multiple alternatives allowed
Other alternatives: text (tx) and numeric (Num) (The rest: See Electronic Form manuals and separate entry SOP)
Langi = Local language

Rule for all categorical answers: DNK tick in under question mark
Rule for all numerical answers: DNK = 99

SECTION I Background

Page a (1)

**1. QUESTION ENGLISH**	**2. ANSWER ENGLISH**	**SKIP INSTRUCTION**	**COLUMN FOR CODING**
1. Site			EF: RB
2. Interviewer	__________		EF: RB<br>4 LETTER CODE;<br>UPPER CASE<br>Choose between drop down list/text<br><br>VN: 01a02<br>**VN ALTERNATIVES: KEEP 4 DIGIT CODE**
3. Date:			EF: Date<br>VN: 01a03
4. Time			EF: Time<br>VN: 01a04
5. GPS	1.<br>2.<br>3.	Optional: Can be deleted as an entry alternative for those who will have this automatic in EF<br>Needs a cable in addition to the GPS<br>Alt recorded in m	EF: GPS<br>VN: 01a05 a1:E/(W) ###°##.###<br>a2: N/S ##°##.###<br>a3: #### (#-####)

READ OUT LOUD:

We come from The Department of Paediatrics and Child Health of Makerere University Medical School. We are conducting a study on Maternal and Child Health. Will you allow me to include you in the study? Thank you so much. Now let's answer the following questions together.

Langi: Consent for screening:
READ OUT LOUD

6. Oral consent for screening given:	1. [_] Yes<br>0. [_] No	**If no,<br>Rule EF:<br>No → Discontinuation from SI<br>- Say thank you and ask for reason for non-participation; fill in separate form**	EF: RB<br>VN: 01a06
7.Main language spoken	1. [_] Langi<br>2. [_] Acholi<br>3. [_] Lugbara<br>4. [_] Alur<br>5. [_] Kumam<br>6. [_] Itesot<br>7. [_] Luganda<br>8.[_]English<br>9.Other[_]<br>Specify……….		EF:RB<br>VN: 01a07
8. Which language is the interview translated into:	1. [_] Langi<br>2. [_] Kumam<br>3. [_] Itesot<br>4. [_] Acholi<br>5. [_] Luganda<br>9. [_] Other specify<br>_______________		EF: Tx<br>VN: 01a08
9. External translator needed:	1. [_] Yes<br>0. [_] No		EF: RB<br>VN: 01a09

	**Do not read out:**<br>10. Note Sub-County/Division	**Do not read out:**<br>11. Note ward/parish= CLUSTER CODE	12. **Read English:** What is the name of your village/cell?<br>**Read Langi:**	**CODING!**
Residence	1. [_] Amach<br>2. [_] Agali<br>3. [_] Adekokwok	1. [_]<br>2. [_]<br>3. [_]<br>4. [_]	1a. [_]<br>1b. [_]<br>2. [_]<br>3. [_]	*EF: RB/ All Mand<br>VN: q 10 Subcounty/Division : 03a10*

	4. [_] Ngetta<br>5. [_] Ogur<br>6. [_] Agweng<br>7. [_] Lira<br>8. [_] Barr<br>9. [_] Aromo<br>10. [_] Adyel<br>11. [_] Ojwina<br>12. [_] Central<br>13. [_] Railway	5. [_]<br>6. [_]<br>7. [_]<br>8. [_]<br>9. [_]<br>10. [_]<br>11. [_]<br>12. [_]<br>13. [_]<br>14. [_]<br>15. [_]<br>16. [_]<br>17. [_]<br>18. [_]<br><br>19. [_]<br>20. [_]<br>21. [_]<br>22. [_]<br>23. [_]<br>24. [_]	4. [_]<br>5. [_]<br>6. [_]<br>7. [_]<br>8a. [_]<br>8b. [_]<br>9. [_]<br>10. [_]<br>11a. [_]<br>11b. [_]<br>12a. [_]<br>12b. [_]<br>13. [_]<br>14a. [_]<br>14b. [_]<br>15a. [_]<br>15b. [_]<br>16a. [_]<br>16b. [_]<br>17a. [_]<br>17b. [_]<br>18a. [_]<br>18b. [_]<br><br>19. [_]<br>20. [_]<br>21a. [_]<br>21b. [_]<br>21c. [_]<br>22a. [_]<br>22b. [_]<br>22c. [_]<br>23a. [_]<br>23b. [_]<br>24a.[_]<br>24b.[_]<br>24c. [_]	*q 11 Ward/Parish: 03a11*<br>*q 12 Village: 03a12*

SECTION II: Current Pregnancy Information
Electronic form Electronic Form p. e (6)

2. Have you had any problems during this current pregnancy?	1. [_] Yes<br>0. [_] No ↓	**SKIP: If no, skip to section III SES, EF page f q. 1**	EF: RB<br>VN: 02e02

3. What problem?	1. [_] Threatened abortion(bleeding)<br>2. [_] Pregnancy Induced Hypertension<br>3. [_] Malaria in pregnancy<br>4. [_] UTI in pregnancy<br>5. [_] Hyperemesis gravidarum<br>6. [_] Anaemia in pregnancy<br>7. [_] High fever with foul vaginal discharge<br>8. [_] Other, specify<br>________________	RULE:<br>**Tick all that apply**	EF: CB<br>VN: 02e03
4. For how long?	ANSWER GIVEN IN:<br>____*(#(#))* (weeks)	RULE:<br>Write answer in months **or** weeks ago. Probe till you get it as exact as possible (< 1 mo=0)	EF: RB<br>EF: Num<br>VN: 02e04
5. Did the problem result in hospitalization?	1.[_] yes<br>0. [_] No		EF: RB<br>VN: 02e05
6. How long was the hospitalisation in days?	………*(#(#))* (days)		EF: Num<br>VN: 02e06

SECTION III Questions on use of clinical/medical services

Electronic form Electronic Form p. j (10)

RULE for section: If she refuses to answer; tick in "Missing" under the question mark alternative

**1. QUESTION ENGLISH**	**2. ANSWER ENGLISH**	**SKIP INSTRUCTION**	**COLUMN FOR CODING**
1. Have you attended any sessions at the antenatal care clinic (ANC)?	1. [_] Yes<br>2. [_] No ↓	**SKIP: If no skip to q. 3**	EF: RB<br>VN: 03j01

2. How many times have you been there in this pregnancy?	__________(#)		EF: Num<br>VN: 03j02
3. Do you use a treated \bed net for yourself?	1. [_] Yes<br>2. [_] No<br>3. [_] Sometimes		EF: RB<br>VN: 03j03
4. Have you been informed about the HIV voluntary counselling and testing (VCT) service?	1. [_] Yes<br>2. [_] No ↓	**SKIP: If no skip to q. 9**	EF: RB<br>VN: 03j04
5. Have you been counselled on HIV?	1. [_] Yes<br>2. [_] No ↓	**SKIP: If no skip to q. 9**	EF: RB<br>VN: 03j05
6. Have you been tested for HIV?	1. [_] Yes<br>2. [_] No ↓<br>3. [_] Do not know	**SKIP: If no skip to q. 9**	EF: RB<br>VN: 03j06
7. Are you willing to tell me the result of your HIV-test?	1. [_] Yes<br>2. [_] No ↓	**SKIP: If no skip to q. 9**	EF: RB<br>VN: 03j07
8. What was the result?	1. [_] Negative<br>2. [_] Positive<br>3. [_] Do not know		EF: RB<br>VN: 03j08
9. If you were given the chance, are you willing to go for voluntary counselling and testing?	1. [_] Yes<br>2. [_] No		EF: RB<br>VN: 03j09

READ OUT LOUD:
Thank you very much for your help! This is a great help for us!
Be free to thank/greet in local language to round off nicely!

A: Survival PLUSS 1 week Questionnaire
List of abbreviations
EF = Electronic form
Radio Button (RB), One alternative only
Check Boxes (CB), Multiple alternatives allowed
Other alternatives: text (tx) and numeric (Num) (The rest: See Electronic Form manuals and separate entry SOP)
Langi = Local language

Rule for all categorical answers: DNK tick in under question mark
Rule for all numerical answers: DNK = 99

SECTION 0 Background

Page a (1)

QUESTION ENGLISH	ANSWER ENGLISH	SKIP INSTRUCTION	6. COLUMN FOR CODING
1. Site			EF: RB
2. Interviewer	_________		EF: RB<br>4 LETTER CODE;<br>UPPER CASE<br>Choose between drop down list/text<br><br>VN: 01a02<br>**VN ALTERNATIVES: KEEP 4 DIGIT CODE**
3. Date:			EF: Date<br>VN: 01a03
4. Time			EF: Time<br>VN: 01a04
5. GPS	1.<br>2.<br>3.	Optional: Can be deleted as an entry alternative for those who will have this automatic in EF<br>Needs a cable in addition to the GPS<br>Alt recorded in m	EF: GPS<br>VN: 01a05 a1:E/(W) ###°##.###<br>a2: N/S ##°##.###<br>a3: #### (#-####)

Enter the following under the Electronic Form type question Label/Title. Remember max. 250 characters.
English: Consent for screening

SECTION I CARE DURING DELIVERY
English:
Now I am going to ask you questions about the child/children you delivered a week ago
Electronic form Electronic Form p. e (5)

QUESTION ENGLISH	ANSWER ENGLISH	RULE/ SKIP INSTRUCTION	COLUMN FOR CODING
1. Where did you deliver from?	[_] Hospital<br>[_] Health center<br>[_] TBA<br>[_] Private clinic<br>[_] Home with health professional<br>[_] Home with TBA<br>[_] Home unattended<br>[_] On route to hospital or health center	1. [_] Eyo<br>2. [_] Pee	EF: RB<br>VN: 01e01
2. What was the mode of delivery	1. [_] Spontenous vaginal delivery<br>2. [_] ceaserian<br>3. [_] Assisted vagina delivery		EF: RB<br>VN: 01e02
3. Who assisted you during delivery?	1. [_] Health professional<br>2. [_] TBA<br>3. [_] Relatives<br>4. [_] Other(specify)<br>…………………..		EF: RB<br>VN: 01e03
4. At what time did labour begin?	[_] Day<br>[_] Night		EF: RB<br>VN: 01e04
5. After how long did delivery take place after onset of labour?	[_] <12 hours<br>[_] >12 hours<br>[_] I don't know<br>[_] Other specify…………………		EF: RB<br>VN: 01e05
6. Where you blood transfused during/after birth?	[_] Yes<br>[_] No		EF: RB<br>VN: 01e06
7. At what time was the baby born?	[_] Day<br>[_] Midday<br>[_] Evening<br>[_] Night		EF: RB<br>VN: 01e07

8. Outcome of Delivery	[_] Single Live birth<br>[_] Multiple Live birth<br>[_] Still birth Born<br>[_] Alive but died		EF: RB<br>VN: 01e08
9. On which surface did the delivery take place?	[_] Labour bed in hospital/health unit with a sheet<br>[_] "Labour bed" at TBA<br>[_] Bare bed<br>[_] Cloth /sheet / makintonsh on floor<br>[_] Bare floor<br>[_]Other(specify)……………		EF: RB<br>VN: 01e09
10. What was used to cut the cord?	1. [_] Razor blade<br>3. [_] Scissors<br>4. [_] Knife<br>5. [_]Don't know<br>6. [_]Other (specify)…………		EF: RB<br>VN: 01e10
11. What was used to tie the cord?	1. [_] Cloth strip<br>2. [_] Thread<br>3. [_] Cord ligature<br>4. [_] Rubber birds<br>4. [_] Don't know<br>5. [_] Other (specify)………….		EF: RB<br>VN: 01e11
12. What was put on the cord to help it heal?	1. [_] Nothing<br>2. [_] Medical drugs<br>3. [_] Powder<br>4. [_] Ash<br>5. [_] Salty water<br>6. [_] Spirit<br>7. [_] Cowdung<br>8. [_] Other (specify)……………….		EF: RB<br>VN: 01e12
13. Did the attendant at delivery counsel you on the following?	1. [_] Breast feeding<br>2. [_] Keeping the baby warm<br>3. [_] Cord care<br>4. [_] Danger signs of the newborn<br>5. [_] Importance of health facility visit<br>6. [_] family planning<br>7. [_] Immunisation<br>8. [_] Vitamin A supplementation<br>9. [_] Other specify…………		EF: RB<br>VN: 01e13

Did you (the mother) experience any of the following signs after delivery?	[_] Abnormal vaginal bleeding<br>[_] loss of consciousness<br>[_] Fever<br>[_] Swelling<br>[_] Convulsions<br>[_]Other specify		EF: CB<br>VN: 01e14
Where you hospitalized	[_] Yes<br>[_] No		EF: RB<br>VN: 01e15
For how long where you hospitalized	………………… days		EF: Num<br>VN: 01e02

SECTION II Delivery experience

Langi: Electronic Form p. e (5)

Now we are going to ask you about your experience during the last delivery.

QUESTION ENGLISH	ANSWER ENGLISH	RULE/ SKIP INSTRUC TION	COLUMN FOR CODING
1. How would you rate your health facility experience for the last pregnancy?	1. [_] Good<br>2. [_] Fair<br>3. [_] Bad		EF:RB<br>VN:02e03
2. Have you/or someone very close to you ever delivered from a TBA?	1. [_]Yes<br>2. [_]No<br>1. [_] Don't know		EF:RB<br>VN:02e02
3. How would you rate their services?	1. [_]Good<br>2. [_]Fair<br>3. [_]Bad		EF:RB<br>VN:02e03
4. How would you compare health facility to TBA in terms of quality of care during birth?	1. [_]Better<br>2. [_]Worse<br>3. [_]Same		EF:RB<br>VN:02e04
5. How would you describe your experience at your last health facility visit during pregnancy or childbirth?	1. [_]very comfortable<br>2. [_]somewhat comfortable<br>3. [_]Uncomfortable<br>4. [_]Very uncormfortable		EF:RB<br>VN:02e05

6. Did you experience any complication during your pregnancy?	[_] Yes<br>[_] No ↓		EF:RB<br>VN:02e06
7. What was the problem?	1. [_] Baby died<br>2. [_] Obstructed labour<br>3. [_] Post partum hemorhage<br>4. [_] Sepsis<br>5. [_] Birth canal trauma<br>6. [_] Fistula<br>7. [_] Intrapartum neonatal complication(Birth asphyxia)<br>8. [_] High fever with foul vaginal discharge<br>9. [_] Other, specify<br>________________	**Rule: Tick all that apply**	EF:CB<br>VN:02e07

Section III: Health facility birth cost

QUESTION ENGLISH	ANSWER ENGLISH	RULE/ SKIP INSTRUCTION	COLUMN FOR CODING
1. During delivery What was the transport cost to and from the health facility	…………………..UGX<br>INAP	**SKIP: If No, skip to q. 3**	EF: Num<br>VN: 03f01
2. What mode of transport did you use to get to the health facility?	1. [_] Private car<br>2. [_] Bus<br>3. [_] Taxi<br>4. [_] Ambulance<br>5. [_] Bicycle<br>6. [_] motorcycle<br>7. [_] On foot<br>8. [_] Other……….	**RULE: Tick off all that apply**<br>**RULE:** Probe if alt. 1 only<br>**PROBE:**<br>Is that all? / Anything else?	EF: CB<br>VN: 03f02
3. What mode of transport did you use to get to get from the health facility to your home?	1. [_] Private car<br>2. [_] Bus<br>3. [_] Taxi<br>4. [_] Ambulance<br>5. [_] Bicycle<br>6. [_] motorcycle<br>7. [_] On foot<br>8. [_] Other……….	**RULE: Tick off all that apply**<br>**RULE:** Probe if alt. 1 only<br>**PROBE:**<br>Is that all? / Anything else?	EF: CB<br>VN:03f03

4. How much did it cost you to buy materials to go with to the place of delivery?	1. [_]Baby clothes ____<br>2. [_]Cotton<br>3. [_]Gauze<br>4. [_]Plastic sheet<br>5. [_]Basin<br>6. [_]mama kit<br>7. [_] Other………Shs<br>Nothing=00	**RULE: Tick off all that apply**<br>**PROBE:**<br>Is that all? / Anything else?	EF:CB<br>VN:03f04
5. How much did you spend during the last pregnancy?	……………..shs<br><br>I don't know =99<br>Nothing=00		EF: Num<br>VN:03f05
6. How much did it cost you to pay the health care provider?	 ………………Shs<br>Nothing = 00<br>Paid in kind = 86<br>(specify)……….<br>DK=<br>Other specify		EF: Num<br>VN: 03f06
7. How far was the place of birth from your home?	…………Km<br>DK=	**SKIP: If no, skip to section IV SES, EF page f q. 1**<br><br>**I mile =1.6km**	EF: Num<br>VN: 03f07
8. Did your husband escort you to the place of delivery?	1. [_] Yes<br>2. [_] No<br>3. Other specify ________		EF: RB<br>VN: 01f08
9. Who in this home decides on where the baby should be delivered from?(DO NOT PROBE TICK ALL MENTIONED)	1. [_] Paternal grandmother<br>2. [_] Maternal grandmother<br>3. [_] Husband<br>4. [_] Mother herself<br>5. [_] Others (specify)…………	RULE:<br>Write answer in months **or** years ago. Probe till you get it as exact as possible (< 1 mo=0)	EF: RB<br>a1: Num<br>a2: Num<br>VN: 01f09

SECTION IV New borne care practices
SKIP instruction: If section II, question 1 = Alternative 2 Skip to question 5

English:
Now I am going to ask you questions about the last child you had
Langi

1. QUESTION ENGLISH	2. ANSWER ENGLISH	5. RULE/ SKIP INSTRUCTION	6. COLUMN FOR CODING
1. Is your baby alive?	1. [_] Yes<br>2. [_] No	**SKIP: If No, skip to q. 3**	EF: RB<br>VN:04e01
3. What is the sex of your baby?	ANSWER GIVEN IN:<br>1. [_] Male<br>2. [_] Female		EF: RB<br>VN:04e02
How many pregnancies have you had?	………………		EF: Num<br>VN:04e03
How many children have you had?	………………….	**SKIP: If no, skip to section IV SES, EF page f q. 1**	EF: Num<br>VN:04e04
How many live children have you had?	_______________		EF: Num<br>VN:04e05
Was the baby weighed immediately after delivery?	1. [_] Yes<br>2. [_] No	RULE: Write answer in months **or** years ago. Probe till you get it as exact as possible (< 1 mo=0)	EF: RB<br>6b: Num<br>VN:04e06
6b. If yes what was the birth weight?	……………(kgs)		
6c.What were you told that weight meant?	[_] Low birth weight<br>[_] Normal weight<br>[_] Large for gestation age<br>[_] Not told		
Did the baby have any problem with breathing or crying immediately after delivery?	1. [_] Yes<br>2. [_] No<br>3. [_] Don't know		EF: RB<br>a3: Tx<br>a5: Tx<br>VN:04e07
Did anyone do the following things on the baby after delivery?	1. [_] Cleaning<br>2. [_] Blow air in the mouth<br>3. [_] Try to bring up the baby's life<br>Other specify………..	**RULE: Tick off all that apply PROBE:**<br>Is that all? / Anything else?	EF:CB<br>VN:04e08

Where was the baby placed immediately after delivery?	1. [_] On the ground<br>2. [_] On the stomach/chest of the mother<br>3. [_] At the side of her mother<br>4. [_] Someone else<br>5. [ ] Other specify)……………		EF: RB<br>VN:04e09
Was the baby dried immediately after delivery?	[_]yes<br>[_]No<br>Don't know		EF: RB<br>VN:04e10
Was the baby wrapped in a cloth after delivery before the placenta came out?	1. [_] Yes<br>2. [_] No<br>3. [_] don't know		EF: RB<br>VN:04e11
What was used to tie the cord?	1. [_] Piece of thread<br>2. [_] Piece of cloth<br>3. [_] Piece of glove<br>4. [_] Other specify		EF: RB<br>VN:04e12
What was used to cut the cord?	1. [_] New razor blade<br>2. [_] Old razor blade<br>3. [_] Pair of scissors<br>4. [_] Spear grass<br>5. [_] Knife<br>4. [_] Other specify……………..		EF: RB<br>VN:04e13
Was the option used for tying the cord already sterilized	1. [_] Yes<br>2. [_] No<br>3. [_] don't know		EF: RB<br>VN:04e14
If yes, how was it sterilized	1. [_] Boiled<br>2. [_] Deeped in spirit<br>3. [ ] Prepacked sterile material<br>4. [_] Other specify…………..		EF: RB<br>VN:04e15
Was anything applied on the cord just after cutting and tying it?	1. [_] yes<br>2. [_] No		EF: RB<br>VN:04e16
If yes by whom?	[_] Mother of baby<br>[_] Father of baby<br>[_] Mother in law<br>[_] Sister<br>[_] grandmother<br>[_] Friend<br>[_]TBA		EF: RB<br>VN:04e17

	[_]Health worker<br>[_] Other(specify)		
What was applied to the cord just after cutting it?	1. [_] Ghee<br>2. [_] Ash<br>3. [_] Medicine to smear<br>4. [_] Dung<br>5. [_] cooking oil<br>6. [_] Salty water<br>7. [_] surgical spirit<br>8. [_] Chlorhexidine<br>9. [_] Others specify…………………		EF: RB<br>VN:04e18
Was anything applied to the skin of the baby?	[_]Yes<br>[_]No		EF: RB<br>VN:04e19
What was applied to the cord/skin just after cutting it?	1. [_] Ghee<br>2. [_] Ash<br>3. [_] Medicine to smear<br>4. [_] Dung<br>5. [_] cooking oil<br>6. [_] Salty water<br>7. [_] surgical spirit<br>8. [_] Chlorhexidine<br>9. [_] Others specify…………………		EF: RB<br>VN:04e20
How long did you take to bath the baby after delivery?	1. [_]In the first 6 hours<br>2. [_]Between 6-24 hours<br>3. [_]Greater than 24 hours		EF: RB<br>VN:04e21
Who bathed the baby?	[_] Mother of baby<br>[_] Father of baby<br>[_] Mother in law<br>[_] Sister<br>[_] grandmother<br>[_] Friend<br>7.[_] Other(specify)		EF: RB<br>VN:04e22
How was warmth maintained after birth?	1. [_] Direct Contact with mother's skin<br>2. [_] Wrapping in cloth<br>3. Electric warmer<br>3. [_] Other(specify)………..		EF: RB<br>VN:04e23
Was head covering done for the baby everytime?	[_]Yes<br>[_]No<br>Don't know<br>other specify	**Head covering includes both capes &**	EF: RB<br>VN:04e24

		cloth/sheet	
Who was the person responsible for that?	[_] Mother of baby<br>[_] Father of baby<br>[_] Mother in law<br>[_] Sister<br>[_] grandmother<br>[_] Friend<br>[_]Health professional<br>[_] Other(specify)		EF: RB<br>VN:04e25
Who did the baby sleep with at night in the first week of life?	1. [_] Slept with the mother<br>2. [_] Slept alone<br>3. [_] the baby with a different person<br>4. Other specify		EF: RB<br>VN:04e26
How long after delivery did you put the baby on breast	1. [_] During the first hour<br>2. [_] After an hour but on the same day<br>3. [_] After one day<br>4. Never		EF: RB<br>VN:04e27
Who was responsible for that decision?	[_] Mother of baby<br>[_] Father of baby<br>[_] Mother in law<br>[_] Sister<br>[_] grandmother<br>[_] Friend<br>[_]Health professional<br>8.[_] Other(specify)		EF: RB<br>VN:04e28
Did you express the first breast milk and discard?	1. [_] Yes<br>2. [_] No<br>3. Other specify<br>_______________		EF: RB<br>VN:04e29
Did you give the baby anything else in the first month apart from breast milk to feed the baby?	1. [_] Yes<br>2. [_] No<br>Don't know		EF: RB<br>VN:04e30
What was given apart from breast milk to feed the baby?	[_]Milk(other than breast milk)<br>[_] Plain water<br>[_]Sugar and salt solution<br>[_]Gripe water<br>[_]Sugar or glucose water<br>[_]Fruit juice		EF: CB<br>VN:04e31

	[_]Infant formula<br>[_]Tea<br>[_]Honey<br>[_]Others (specify)……		
On the first day after births, did any health provider examine you (the mother)?	1. [_] Yes<br>2. [_] No		EF: RB<br>VN:04e32
Did any health worker visit you to provide care or advice in the first month after delivery?	1. [_] Yes, health worker<br>2. [_] Yes, village health team /CHW<br>1. [_] Yes, TBA<br>3. [_] No<br>4. Don't know<br>Other specify		EF: RB<br>VN:04e33
After delivery, did you ever take your child to a health facility for check up?	1. [_] Yes<br>2. [_] No		EF: RB<br>VN:04e34
How old in days was the baby at the time of the first check at a healthy facility or home?	………………………….<br>(weeks)		EF: Num<br>VN:04e35
How many times did your baby have a check up at the health facility or home?	………………………..		EF: Num<br>VN:04e36
During the baby's check, was the following done?	1. [_]Vitamin A<br>2. [_] Immunisation for the baby?		EF: RB<br>VN:04e37
Have you ever fed the baby with a bottle?	1. [_] Yes<br>2. [_] No<br>3. Don't know		EF: RB<br>VN:04e38
Where you ever advised on how to breastfeed the baby?	1. [_]Yes<br>2. [_]No<br>3. Don't know		EF: RB<br>VN:04e39
Do people who regularly care for the baby wash their hands before handling the baby?	1. [_]Yes, all the time<br>3. [_]Yes, Sometimes<br>3. No		EF: RB<br>VN:04e40
What do they use to wash their hands?	1. [_] Water only<br>2. [_] Water and soap<br>3. [ ] Sanitizer<br>4.[_] Other (specify)….........		EF: RB<br>VN:04e41
New born babies often get ill and some illnesses are serious to their lives in the first month of life. Which signs may show that a new	1. [_]Convulsions or fits<br>2. [_]Inability to feed adequately or cessation of suckling<br>3. [_]Lethargy(very	**DO NOT PROBE. CIRCLE ALL MENTIONE**	EF: CB<br>VN:04e42

born's health may be in danger?	weak)<br>loss of consciousness<br>4. [_]Redness of the umbilicus extending to the skin of the abdomen<br>5. [_]Persistent vomiting abdominal distention<br>6. [_]Difficulty breathing<br>7. [_]Fever<br>unusually cold body temperature<br>8.<br>[_]Other(specify)………<br>……….	**D**	
Has your child had any of the following signs?	1. [_]Convulsions or fits<br>2. [_]Inability to feed adequately or cessation of suckling<br>3. [_]Lethargy(very weak)<br>loss of consciousness<br>4. [_]Redness of the umbilicus extending to the skin of the abdomen<br>5. [_]Persistent vomiting abdominal distention<br>6. [_]Difficulty breathing<br>7. [_]Fever<br>unusually cold body temperature<br>8. [_]<br>Other(specify)…………<br>…….		EF: CB<br>VN:04e43
**Diarrhoea**			
<NAME>diarrhoea?(more than three watery stools, not typically breast milk stools, in <24hours)	1. [_] Yes<br>2. [_]No ↓		EF: RB<br>VN: 05f01
Did <NAME> pass any watery stools?	[_] Yes<br>[_] No		EF: RB<br>VN: 05f02
The day <NAME> had most loose or watery stools, how many loose or watery stools did <NAME> pass?	……………(##)		EF: Num<br>VN: 05f03
Did any of the stools contain blood?	[_] Yes<br>[_] No		EF: RB<br>VN: 05f04

Were the stools of different consistency than before <NAME> fell ill with diarrhoea?	[_] Yes<br>[_] No		EF: RB<br>VN: 05f05
Did the illness interfere with <NAME>'s ability to drink or eat?	[_] Yes<br>[_] No		EF: RB<br>VN: 05f06
Did you seek treatment for < Name>	[_] Yes<br>[_] No↓		EF: RB<br>VN: 05f07
Where did you go?	[_] Relatives and friends<br>[_] Traditional healer<br>[_] Drug shop/Pharmacy<br>[_] Government or private clinic/surgery/community health centre including general practitioner<br>[_] The emergency/outpatient department of a hospital<br>[_] Other, specify…………		EF: CB<br>VN: 05f08
Was the child admitted to a hospital?	[_] Yes<br>[_] No ↓		EF: RB<br>VN: 05f09
Please give the name of the hospital?	[_]<br>[_]<br>[_]<br>[_]<br>[_]<br>[_]<br>[_]<br>[_]		EF: RB<br>VN: 05f10
Was this the nearest health unit?	[_] Yes<br>[_] No		EF: RB<br>VN: 05f11
Why did you go there?	[_] Health services better than at the nearest health unit<br>[_] Transport was available<br>[_] The nearest health unit is more expensive than the other I went to<br>[_] I wanted to go to the biggest hospital I can afford		EF: CB<br>VN: 05f12

	[_] I do not trust the people at the nearest health unit<br>[_] Other, specify		
ALT1:How many days did the diarrhoea last/<br>ALT 2: How many days has the diarrhoea lasted?	[_]Finished: Duration in days…………….<br>[_] Not finished: Duration in days:………		EF: Num<br>VN: 05f13
Is your child still alive?	[_] yes<br>[_] No↓		EF: RB<br>VN: 10f14
Has your child changed in any way after that illness?	[_] yes<br>[_] No ↓		EF: RB<br>VN: 05f15
In which way?	[_] stopped breast feeding<br>[_] Became smaller<br>[_] Other specify………		EF: RB<br>VN: 05f16
Who in this home decides on where the baby should be treated from?	[_] Paternal grandmother<br>[_] Maternal grandmother<br>[_] Husband<br>[_] The mother her self<br>[_] Other (specify)………		EF: RB<br>VN: 05f17
1. During the last two weeks that ended yesterday morning, did <NAME> have cough?	1. [_] Yes<br>2. [_] No		EF: RB<br>VN: 05g01
2. During the last two weeks that ended yesterday morning, did <NAME> have fast or difficult breathing?	1. [_] Yes<br>2. [_] No ↓		EF: RB<br>VN: 05g02
3. Did the illness interfere with <NAME>'s ability to drink or eat?	1. [_] Yes<br>2. [_] No		EF: RB<br>VN: 05g03
4. Was <NAME> admitted to a hospital for the illness?	1. [_] Yes<br>2. [_] No ↓		EF: RB<br>VN: 05g04
5. Please give name of hospital?	1. [_]<br>2. [_]<br>3. [_]<br>4. [_]<br>5. [_]<br>6. [_]<br>7. [_]<br>8. [_]<br>9. [_]		EF: RB<br>VN: 05g05

	10. [_]<br>11. [_]<br>12. [_]<br>_________________		
6. Was this the nearest health unit?	1. [_] Yes<br>2. [_] No ↓		EF: RB<br>VN: 05g06
7. Why did you go there?	1. [_] Health cervices better than at the nearest health unit<br>2. [_] Transport was available<br>3. [_] The nearest health unit is more expensive than the one I went to<br>4. [_] I wanted to go to the biggest hospital I can afford<br>5. [_] I do not trust the people at the nearest health unit<br>6. [_] Other, specify		EF: RB<br>VN: 05g07
8. During this period of illness you have described, did you change the way you were feeding your child in any way?	1. [_] Yes<br>2. [_] No ↓		EF: RB<br>VN: 05g08
9. In which way?	1. [_] Stopped breast feeding<br>2. [_] Stopped non-human milk<br>3. [_] Stopped other liquids<br>4. [_] Stopped solid foods<br>5. [_] Only breast fed at night<br>6. [_] Began giving other liquids<br>7. [_] Began giving solid foods<br>8. [_] Other, specify		EF: CB<br>VN: 05g09
10. During the period of illness did you feed your	1. [_] More often<br>2. [_] More seldom than		EF: RB<br>VN: 05g10

baby more often, more seldom than or just as often as before the illeness started?	before the illness started<br>3. [_] Did not change feeding frequency.		
Did the child experience any vomiting (more than three episodes of forceful vomiting in <2hrs)?	[_] Yes<br>[_] No		EF: RB<br>VN: 05h01
Did the child experience any abnormal bEFaviour like excess irritability or abnormal sleepiness?	[_]Yes<br>[_]No		EF: RB<br>VN: 05h02
Did the child experience feeding difficulty i.e unable to feed as well as usual or expected	[_]Yes<br>[_]No		EF: RB<br>VN: 05h03
Did this child experience any convulsion?	[_]Yes<br>[_]No		EF: RB<br>VN: 05h04
Did this child have any yellowing of eyes?	[_]Yes<br>[_]No		EF: RB<br>VN: 05h05
Did the child experience any swelling or pus discharge from the eyes?	[_]Yes<br>[_]No		EF: RB<br>VN: 05h06
Did the child experience any fast breathing?	[_]Yes<br>[_]No		EF: RB<br>VN: 05h07
Did the child experience any noisy breathing?	[_]Yes<br>[_]No		EF: RB<br>VN: 05h08
Did the child experience any chest indrawing?	[_]Yes<br>[_]No		EF: RB<br>VN: 05h09
Did the child's gums or tongue turn dark blue in colour?	[_]Yes<br>[_]No		EF: RB<br>VN: 05h10
Did the child experience any difficulty in arousal on stimulation?	[_]Yes<br>[_]No		EF: RB<br>VN: 05h11
Did the child experience any inability to attach or suck adequately at breast?	[_]Yes<br>[_]No		EF: RB<br>VN: 05h12
Did the child ever become uncounscious?	[_]Yes<br>[_]No		EF: RB<br>VN: 05h13
Did the child experience any pus over the umbilical area?	[_]Yes<br>[_]No		EF: RB<br>VN: 05h14
Did the child ever become rigid?	[_]Yes<br>[_]No		EF: RB<br>VN: 05h15
Did the child ever become febrile?	[_]Yes<br>[_]No		EF: RB<br>VN: 05h16
Did the child ever become	[_]Yes		EF: RB

unusually cold?	[_]No		VN: 05h17
Did you seek treatment for the childs illness?	[_]Yes<br>[_]No		EF: RB<br>VN: 05h18
Where did you go?	[_]Govt hospital<br>[_]Health center<br>[_]Other public (specify)<br>[_]Private hospital/clinic<br>[_] Drug shop<br>[_]Other/ traditional healer home		EF: RB<br>VN: 05h19
If more than one, in what order?	……………………		EF: RB<br>VN: 05h20

Baseline survey
SECTION VI: Hospitalizations

QUESTION ENGLISH	ANSWER ENGLISH	SKIP INSTRUCTION	COLUMN FOR CODING
1. Since birth has <NAME> ever been admitted to hospital?	1. [_] Yes<br>2. [_] No ↓	**SKIP: If no, skip to S V**	EF: RB<br>VN: 06l01
2. How many times has <NAME> been admitted to hospital? B	_____ (#(#))		EF: Num<br>VN: 06l02
3. How old in weeks was your baby (each time) when he/she was in hospital?	1. [_] 1st time _______<br>2. [_] 2nd time _______<br>3. [_] 3rd time _____<br>4. [_] 4th time _______<br>5. [_] 5th time _______<br>6. [_] 6th time _______<br>7. [_] 7th time _______<br>8. [_] 8th time _______	**RULE: Tick off all that apply**	EF: CB<br>a1: Num<br>a2: Num<br>a3: Num etc.<br><br>VN: 06l03

4. For how many days was <NAME> (each time) in hospital?	1. [_] 1st time _______<br>2. [_] 2nd time _______<br>3. [_] 3rd time _____<br>4. [_] 4th time _______<br>5. [_] 5th time _______<br>6. [_] 6th time _______<br>7. [_] 7th time _______<br>8. [_] 8th time _______		EF: CB<br>a1: Num<br>a2: Num<br>a3: Num etc.<br><br>VN: 06l04
What was the reason <NAME> was in the hospital each time: Ask if they have medical record n read off		**RULE: ENTER THE CORRECT NUMBER FROM THE LIST BELOW**<br>1 = Diarrhoea<br>2 = Pneumonia/ "Cough and difficult breathing"<br>3 = Malaria<br>4 = Accident<br>5 = Specify what ____________	EF: CB<br>a1: Tx<br>a2: Tx<br>a3: Tx etc.<br>Allows for DNK for alternatives<br><br>VN: 06l05

SECTION VII: Baby's born weighing less than 2.5kg at birth

Electronic formElectronic Form p. l (12)

**QUESTION ENGLISH**	**ANSWER ENGLISH**	**SKIP INSTRUCTION**	**COLUMN FOR CODING**
1. Was your baby born at term?	1. [_] Yes<br>2. [_] No ↓<br>96. [_] Don't know	**SKIP: If no, skip to S V**	EF: RB<br>VN: 07l01
2. How many weeks before the expected date was (name) born?	_____ (#(#))		EF: Num<br>VN: 07l02
3. Did you make extra visits to the health facility because your baby was small?	1. [_]Yes<br>2. [_]No	**RULE: Tick off all that apply**	EF: CB<br>a1: Num<br><br>VN: 07l03

4. Were you given any information on how to care for small babies?	1. [_] Yes<br>2. [_] No<br>Don't know		EF: CB<br>a1: Num<br>a2: Num<br>a3: Num etc.<br><br>VN: 07l04
5. What were you told?	1. [_] more frequent breastfeeding<br>2. [_] skin to skin care<br>3. [_] delay first birth for a week or longer<br>4. [_] take baby to health facility<br>5. [_] Extra PNC visits<br>6. [_] Other (specify)…….<br>7. [_] Don't remember?<br>7. [_] 7th time _______<br>8. [_] 8th time _______	____________	EF: CB<br>a1: Tx<br>a2: Tx<br>a3: Tx etc.<br>Allows for DNK for alternatives<br><br>VN: 07l05<br>**Tick off all that apply**
6. Do you feel the information you received was useful to help you care for the baby well?	1. [_]Yes, very useful<br>2. [ ]Yes, somewhat useful<br>3. [_]No, not useful		EF: RB<br>VN: 07l06
7. Were you able to do all that you were told?	1. [_]Yes<br>2. [_]No		EF: RB<br>VN: 07l07
8. If no why?	Think of some viable options and then add other specify		EF:CB<br>VN:07l08
9. EFe was your baby taken to a health facility for special care?	1. [_]Yes<br>2. [_]No		EF:CB<br>VN:07l09
10. Did (Name) receive Kangaroo Mother Care (KMC) at a health facility?	1. [_]Yes<br>2. [_]No		EF:CB<br>VN:07l10
11. When did KMC start?	……(hours)		EF: Num<br>VN: 07l11
12. For how long did (Name) receive KMC in the health facility?	…….. (days)		EF: Num<br>VN: 07l12

13.How long did KMC continue at home?	…….(days)		EF: Num<br>VN: 07l13
