# Supplementary material for: Assessing a bundle of peer counseling, mobile phone messages, and mama kits in promoting timely initiation of and exclusive breastfeeding in Uganda: A cluster randomized controlled study
Source: PLoS One. 2025 Jan 24;20(1):e0317200. doi: 10.1371/journal.pone.0317200 (PMC11761178; doi:10.1371/journal.pone.0317200)
Supplement: S5 File — (PDF) [file pone.0317200.s005.pdf]

Map of study area for the study

Assessing a bundle of peer counseling, mobile phone messages, and mama kits in promoting timely initiation of and exclusive breastfeeding in Uganda: a cluster randomized controlled study

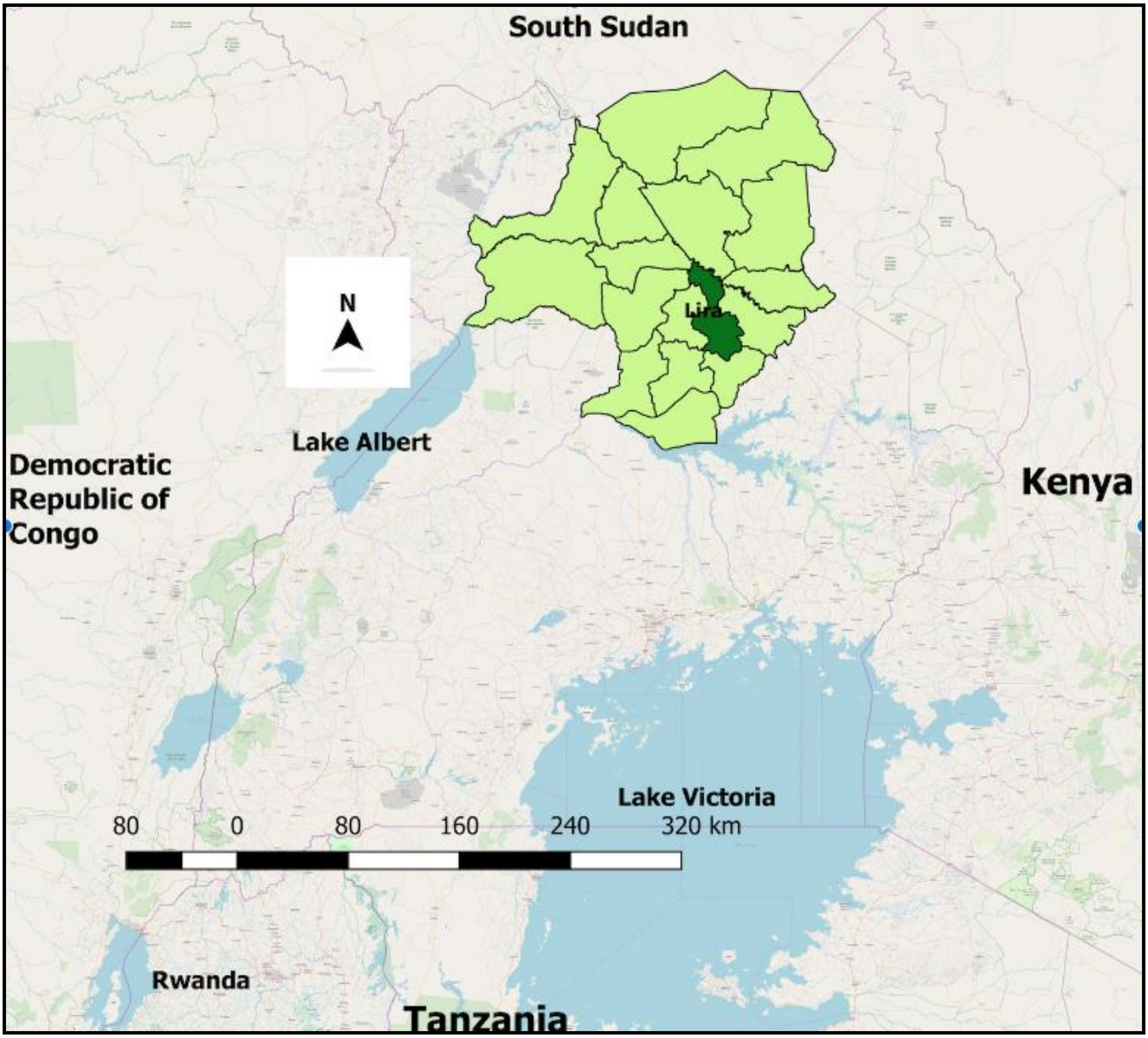

Map of Uganda showing the location of Lira District (dark green) and Northern Uganda (light green)

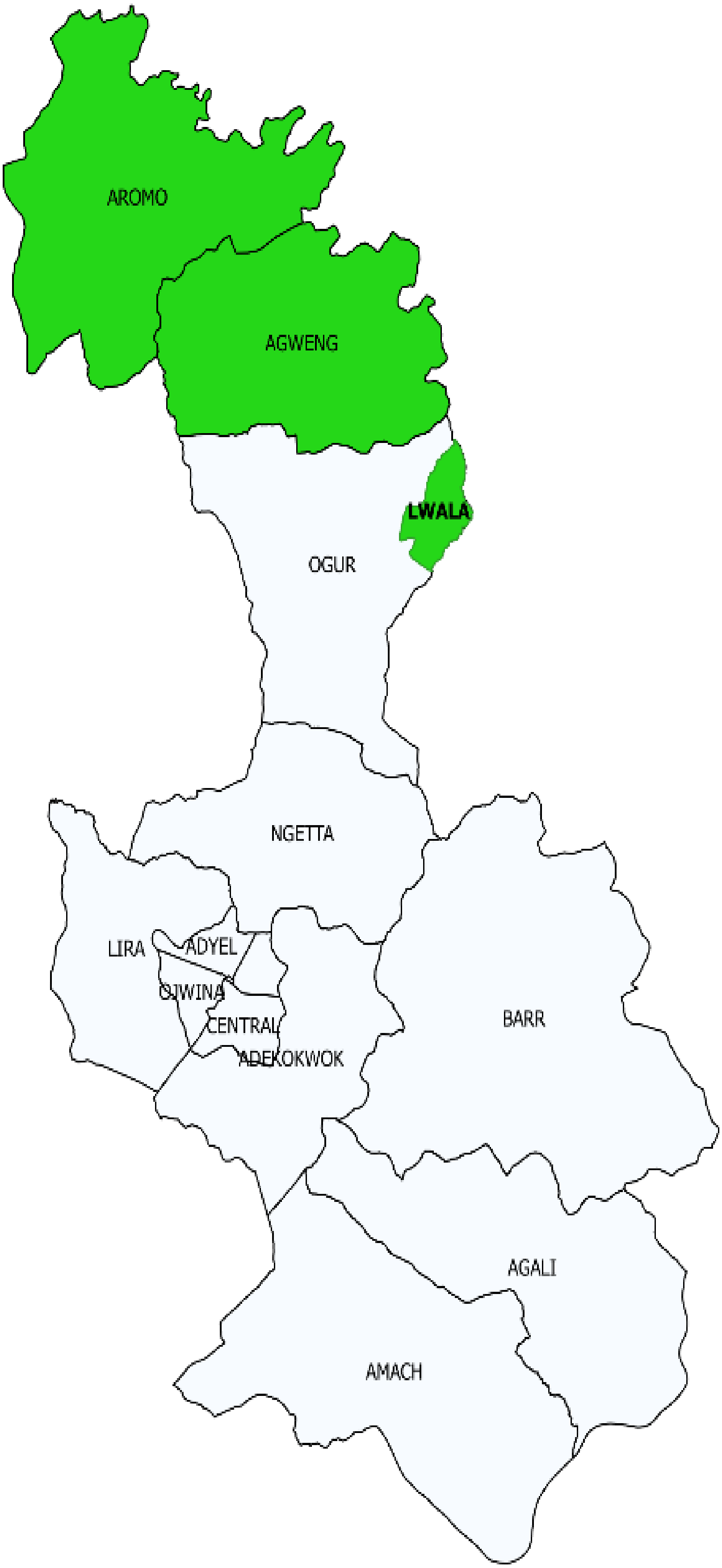

Map of Lira District showing location of the Survival Pluss Intervention (green area)

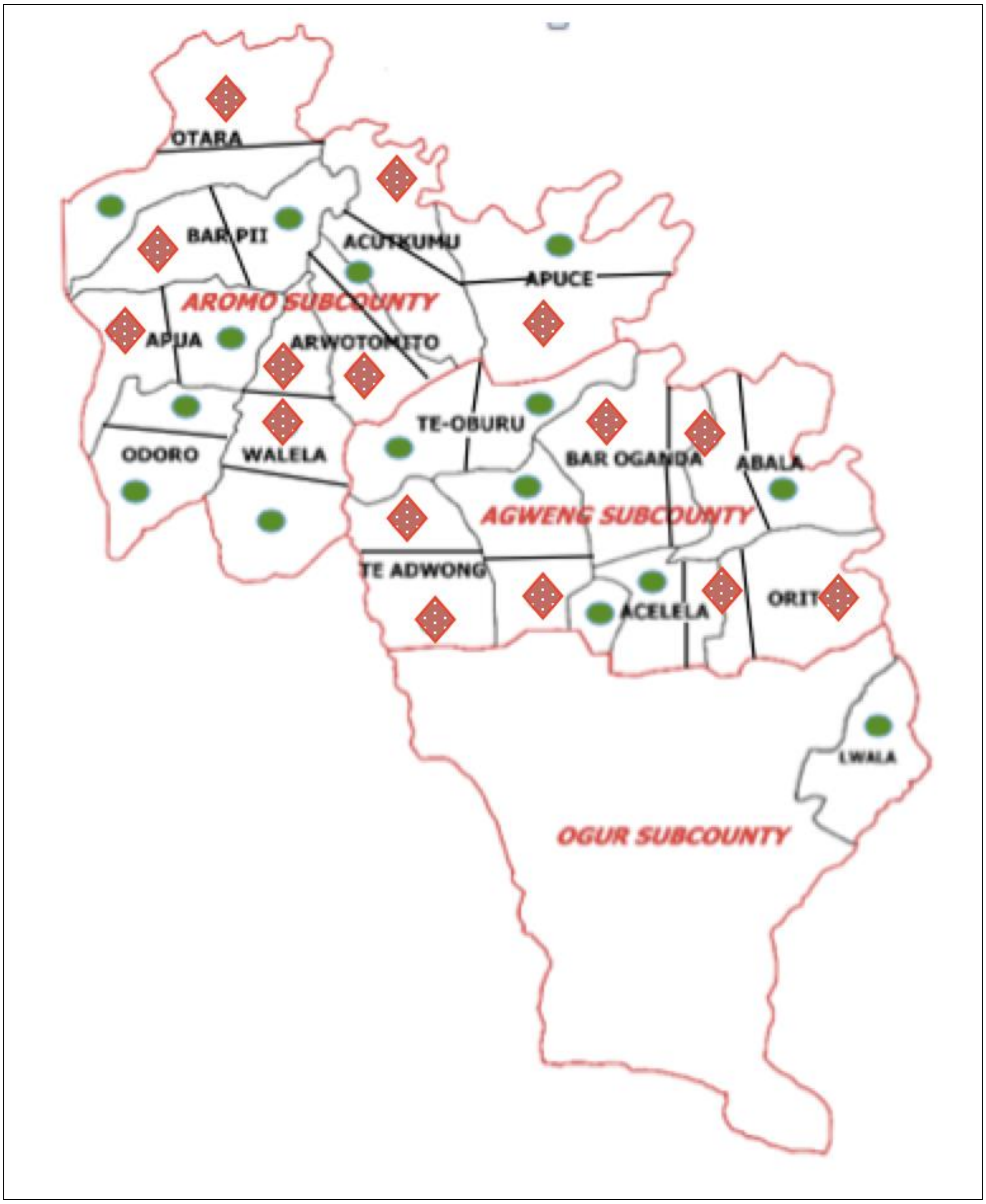

Map of Aromo Agweng and Agali showing intervention (green dots) and control (red diamonds) clusters
