# Supplementary material for: Assessing a bundle of peer counseling, mobile phone messages, and mama kits in promoting timely initiation of and exclusive breastfeeding in Uganda: A cluster randomized controlled study
Source: PLoS One. 2025 Jan 24;20(1):e0317200. doi: 10.1371/journal.pone.0317200 (PMC11761178; doi:10.1371/journal.pone.0317200)
Supplement: Attachment — Submitted filename: Plos response letter_240524.docx [file pone.0317200.s006.docx]

Dear Prof Jeanelle Pauline P. Cupino,

Editor,

PLOS ONE Journal

**Ref: Response to Edits requested on my submission PONE-D-24-18566 - [EMID:ec93ff24fa5296d1]**

In reference to this subject, please find attached a point by point response to the edits requested to the submission of our manuscript titled **“Assessing a bundle of peer counseling, mobile phone messages, and mama kits in promoting timely initiation of and exclusive breastfeeding in Uganda: a cluster randomized controlled study.”**

| Comment | Response |
| --- | --- |
| 1) Please upload a copy of your trial study protocol as a supporting information file. By the study protocol, we mean the complete and detailed plan for the conduct and analysis of the trial that the ethics committee approved before the trial began. Please send this in the original language. If this is in a language other than English, please also provide a translation. Your study protocol will be made available to the editors and reviewers, and will be published as supporting information with your manuscript if accepted for publication. | This has been provided |
| 2) Please upload a copy of the original ethics approval letter as obtained from Regional Committees for Medical and Health Research Ethics (REK VEST 2017/2079), as required by question 1 of the human subject research checklist. If the original letter is not in English, please also provide an English translated version in the supporting file. | This has been provided |
| 3) During our evaluation of the documents provided, we noted that your ethics approval letters (SOMREC:REF 2015-121 and UNCST: SS3954) did not cover the entire date range for participant recruitment. Before we can proceed further with the submission, please provide the ethics approval extension documents for the study. If the document is in another language, please also provide an English translation. Please note that if this document is not included when your manuscript is resubmitted, it may be rejected." | These have been provided.  For the Ugandan Ethical Approval; we have provided 3 renewal documents. However, we have failed to trace one document that covers the period September 2018 to February 2019. These documents were only provided physically at the time of approval and many of these offices were disrupted by the COVID-19 lockdowns, with change in staff and location.  Kindly note that the Norwegian ethical documents cover this period. |

Kind regards

David Mukunya
