# Supplementary material for: Assessing a bundle of peer counseling, mobile phone messages, and mama kits in promoting timely initiation of and exclusive breastfeeding in Uganda: A cluster randomized controlled study
Source: PLoS One. 2025 Jan 24;20(1):e0317200. doi: 10.1371/journal.pone.0317200 (PMC11761178; doi:10.1371/journal.pone.0317200)
Supplement: Attachment — Submitted filename: Plos__one_response letter_121024_final.docx [file pone.0317200.s007.docx]

Dear Editor,

Trhas Tadesse Berhe, PhD
Academic Editor
PLOS ONE

Thank you for the opportunity to have our manuscript PONE-D-24-18566

“Assessing a bundle of peer counselling, mobile phone messages, and mama kits in promoting timely initiation of and exclusive breastfeeding in Uganda: a cluster randomized controlled study” reviewed.

We are extremely pleased with the thorough work by the editor and the reviewers, and we want to express our gratitude for your attention to and effort on our manuscript. We have now gone through all the comments and made the necessary amendments of the text. Please find our line-by-line responses to the reviewers’ comments below.

| Comment | Response | Page and line number |
| --- | --- | --- |
| **Editor’s comments** |  |  |
| Ensure all abbreviations are explained, and figures/tables are clearly labelled.  Consistent statistical language will strengthen the manuscript. | Done. |  |
| Consider adding broader context on public health interventions, especially regarding reproductive rights and maternal autonomy. | We have edited our discussion to include a broader context on maternal autonomy. | Page 21:  Lines 408 to line 415 |
| Ensure your dataset is complete and aligned with the manuscript. Include a codebook for [clarity as](http://clarity.as/) a supplementary file | We have attached a complete data set and a code book as supplementary files. |  |
| Include more specific information about the intervention content, including the mobile messages and peer counselling components. This will enhance reproducibility and allow others to better understand and replicate the study. | We have clarified the peer counselling and mobile messages components and added the messages. Below are some of the messages we have added. *“Welcome to Survival Pluss SMS service! We will be sending a weekly message about your pregnancy”,” Make a plan with your family to put your new baby to the breast in the first hour. Your creamy first milk will protect him from illness”, “Your first milk is best for your baby. Let your partner know you want to breastfeed your baby within the first hour. He can support you”, “Breastfeeding helps to reduce your bleeding after birth. Go to the clinic if your bleeding becomes heavy, clotted, smelly or you feel faint”* | Page 7  Line 157  To line 162  Page 8  Line 175 to line 181 |
| Justify the choice of statistical models used in your analysis. Additionally, consider mentioning any sensitivity analyses conducted to assess the robustness of your findings. | We used a generalized estimation equation (GEE) with a log/identity link and binomial family. The choice of GEE was because we had clustered data (at one level), and the outcome was binary, and we were interested in marginal (population) effects. In such situations, Zeger (1) recommends the use of a GEE model. We used a log link to get prevalence ratios, and an identity link to get prevalence differences. Another advantage of using a GEE is that we can specify the correlation structure. We did sensitivity analyses to assess whether the intervention effect on timely breastfeeding was modified by the place of birth. This analysis was planned *a posteriori* to explore whether there was stronger evidence of the effect of the intervention on timely breastfeeding among those who gave birth at a health facility. To quantify any biological interaction between place of birth and the intervention, we estimated the absolute excess risk due to interaction by using Stata’s icp command to fit a log-binomial model and we adjusted for clustering as shown in the Stata code below.  *icp, show: binreg timely_breastfeeding intervention place_of_birth, nolog cformat(%7.3f) vce(cluster cluster) rr* |  |
| The methodology section is detailed, but there is a need for more information on how participant characteristics (e.g., presence of electricity and mobile phones) were controlled for in the analysis. Please provide a more thorough explanation of the statistical methods used to account for these socio-economic factors. | We adjusted for electricity and mobile phone differences in the intervention and control group in the generalized estimation equation (GEE) models. These results are presented in Table 3. |  |
| The results are presented clearly, but the interpretation of the findings could be strengthened. Specifically, discuss why the intervention did not significantly impact timely breastfeeding initiation despite improvements in exclusive breastfeeding rates.  Are there other factors that might have influenced these outcomes? | Health workers and the place of birth had a role to play to play in initiation of breast feeding and this could have affected the results.  However, we did not assess for access health workers’ knowledge and practices regarding timely breastfeeding initiation and health facility barriers regarding timely breastfeeding initiation, we have included this in our limitations. | Page 24  Line 474 to line 476 |
| The figures and tables are informative but might benefit from additional explanatory notes. Ensure that all tables are referenced in the text and that figures are accompanied by captions that clearly describe what is being presented | This has been addressed. We have included all tables in the text.  Table 1  Table 2  Table 3  Table 4  Figure 1  Figure 2  Figure 3 | Page 15  Page 17  Page 18  Page 20  Page 14  Page 20  Page 20 |
| The paper is generally well-written, but there are a few areas where language and grammar could be improved for clarity. For instance, review the use of passive voice and ensure consistent terminology throughout the paper | Done. |  |
| **Journal requirements** |  |  |
| 1.Please ensure that your manuscript meets PLOS ONE's style requirements, including those for file naming. | Done. |  |
| 2. Thank you for stating the following financial disclosure:  We acknowledge the Survival Pluss project (no. UGA-13-0030) at Makerere University. Survival Pluss project was funded under NORHED by Norad; Norway.    Please state what role the funders took in the study.  If the funders had no role, please state: "The funders had no role in study design, data collection and analysis, decision to publish, or preparation of the manuscript."  If this statement is not correct you must amend it as needed.  Please include this amended Role of Funder statement in your cover letter; we will change the online submission form on your behalf. | Our financial disclosure statement now reads “The funders had no role in study design, data collection and analysis, decision to publish, or preparation of the manuscript.” | Page 25 Line 495 to line 496 |
| 3. We note that you have provided funding information that is not currently declared in your Funding Statement. However, funding information should not appear in the Acknowledgments section or other areas of your manuscript. We will only publish funding information present in the Funding Statement section of the online submission form.  Please remove any funding-related text from the manuscript and let us know how you would like to update your Funding Statement.  Please include your amended statements within your cover letter; we will change the online submission form on your behalf. | We have removed the funding statement from the acknowledgments section | Page 25  Line 481 |
| 4. Your ethics statement should only appear in the Methods section of your manuscript. If your ethics statement is written in any section besides the Methods, please move it to the Methods section and delete it from any other section. Please ensure that your ethics statement is included in your manuscript, as the ethics statement entered into the online submission form will not be published alongside your manuscript. | Done. | Page 9  Line 194 to line 207 |
| 5. Please review your reference list to ensure that it is complete and correct. If you have cited papers that have been retracted, please include the rationale for doing so in the manuscript text, or remove these references and replace them with relevant current references. Any changes to the reference list should be mentioned in the rebuttal letter that accompanies your revised manuscript. If you need to cite a retracted article, indicate the article’s retracted status in the References list and also include a citation and full reference for the retraction notice. | This has been addressed. Our reference list does not contain any retracted papers  We had two duplicates.  1.Citation 33 and citation 52  2.Citation 44 and 46  Therefore, the total number of references has reduced from 52 to 50 |  |
| Reviewer 1 |  |  |
| Figure 1 Trial profile: Please add which group is the intervention group. Please also explain LTFU, EBF, and EBI. | This has been addressed. We have written the full forms of all the acronyms. | Page 14  Line 321 |
| Line 293: Is it a supplementary table? | We have clarified and included table 1 as an independent table in the manuscript, | Page 15 |
| Table 1: You don’t need to report the No, when you report the Yes (electricity, mobile phone, antenatal care visits, sex, LBW). | We have edited the table and only reported the yes responses | Page 15 and page 16 |
| Table 2, 3 and 4: Please explain the abbreviation in the tables (even though it is explained in the text) (BFI, BF, EBI, EBF) | This has been addressed. We have written the full forms of all the acronyms. | Page 16, 17,18 and 19 |
| Line 326-328: Please optimize the sentence for better reading. (e.g: …; fewer mothers (32%, n=555) reported they themselves were responsibe for the timing of the first feed). | We have edited the sentence to provide clarity. | Page 19  Line 362 to line 363 |
| Please discus in the limitation section the 55% coverage of mobile phones when bundle included mobile phone messages. | We have added this as a limitation. | Page 23  Line 460 to line 461 |
| Line 338: The title of Table 4 is beneath the table (it is above the other three tables). | We have edited the title for table 4 and placed it above the table. | Page 20  Line 376 to line 377 |
| Figure 2: Please name “0” and “1” (Motherdecides).  Make sure the figure has a title. | Done. | Page 20  Line 380 |
| **Reviewer 2** |  |  |
| In the abstract, rephrase the sentence containing the words "equally likely" to reflect the more statistically accurate phraseology provided in the following statement. The proportion of participants in the intervention initiating breastfeeding within the first hour of life did not significantly differ from that in the control group. | We have edited this section accordingly. | Page 2  Line 46 to line 48 |
| 2- Supplementary Table 1: Provide p-values comparing the control to the intervention arm. | We were instructed to follow the consort statement in writing this manuscript.  The consort statement categorically discourages reporting P-values and any inferential statistics in Table 1.  Below are the some references and explanations to the thinking behind this recommendation  1.M J Knol, R H H Groenwold, D E Grobbee, P-values in baseline tables of randomised controlled trials are inappropriate but still common in high impact journals, European Journal of Preventive Cardiology, Volume 19, Issue 2, 1 April 2012, Pages 231–232, <https://doi.org/10.1177/1741826711421688>  2.Moher D, Hopewell S, Schulz KF, Montori V, Gøtzsche PC, Devereaux PJ, Elbourne D, Egger M, Altman DG. CONSORT 2010 explanation and elaboration: updated guidelines for reporting parallel group randomised trials. Bmj. 2010 Mar 24;340.  (2, 3). |  |
| 3- Table 2: State the statistical testing method used to estimate the 95% confidence intervals. | We used Huber/white/sandwich variance estimation. We have added this in the manuscript. | Page 13 Line 298 to line 299 |
| 4- The standard statistical term for average is mean. | We have edited the word ‘average’ in table 4 to ‘mean’ | Page 20 |
| Reviewer 3 |  |  |
| Abstract |  |  |
| Line 30 - if this is the target- you may want to state what the current norm is. | We have included this in the introduction section of the main manuscript.  The word limit for the abstract section doesn’t not allow us add any extra information. | Page 4  Line 71 to line 72 |
| Introduction |  |  |
| Line 69- these numbers are different to what you have quoted in your abstract | Thank you for highlighting this, the error was in the abstract section, which we have we have edited accordingly. | Page 2  Line 30 to line 32 |
| Line 81- it is not just the child that initiates or controls breastfeeding, i think the author is trying to say, children who are breastfed early, not 'initiate'. | We have edited the sentence accordingly. | Page 4 Line 82 |
| Results |  |  |
| Line 287-288- Is there any explanation as to why mothers in the intervention arm were far more likely to have electricity than the control arm or is this by chance? | Thank you for this observation,  We think this happened by chance. |  |
| **Reviewer 4** |  |  |
| Introduction |  |  |
| The first part of the introduction effectively highlights the significance of this research (lines 68 to 113). However, the second part (lines 114 to 137) would benefit from revisions for clarity, with some elements better suited for the Methods section. The introduction should conclude with a clear statement of the paper's objective. | We have revised the second section of the introduction accordingly. | Page 5 to page 6  Line 116 to line 121 |
| Methods |  |  |
| However, the “sample size” section (lines 193-204) is somewhat unconvincing, as it’s difficult to believe that the sample size was estimated without a specific target below 1,877 participants. I recommend stating upfront that the sample size was calculated based on the primary objective (rather than mentioning this at the end of the section). | We have edited this section accordingly. | Page 9  Line 209 to line 210 |
| Additionally, it would be more transparent to explain that the authors used Stata IC to verify what could be achieved with the given sample size. | We have edited this section accordingly. | Page 9  Line 211 to line 213 |
| The authors reference a “baseline survey” (lines 130 & 197). Could you provide a brief description of this survey? | We conducted a survey in 2016 of 930 children under the age of 2 years in Lira district, northern Uganda. Mothers of the children were interviewed and data was collected on mobile phones using Open Data Kit software (https://opendatakit.org).  Almost half 48.2% of the mothers delayed initiation of breastfeeding.  We have provided a citation to the published manuscript of this survey.  Mukunya D, Tumwine JK, Nankabirwa V, Ndeezi G, Odongo I, Tumuhamye J, Tongun JB, Kizito S, Napyo A, Achora V, Odongkara B. Factors associated with delayed initiation of breastfeeding: a survey in northern Uganda. Global health action. 2017 Jan 1;10(1):1410975. | Page 9 Line 213 to line 216 |
| Three outcomes were investigated: (1) initiation of breastfeeding within the first hour after birth, (2) exclusive breastfeeding in the 24 hours before the visit 28 days after birth, and (3) exclusive breastfeeding during the first 28 days of life. While the first outcome is detailed (lines 207-214), the other two are only briefly mentioned (lines 214-217). These outcomes should be expanded upon with clear definitions in the Methods section, along with information on data collection: When did the 28-day visit actually take place? How were the two outcomes measured? | We have edited our outcome section to clarify how the 24hour recall was done and 28day visit was conducted. | Page 11  Line 241 to line 246 |
| In the Methods section, please clarify the “socio-economic status quintiles” variable. Was it based solely on wealth? How was wealth assessed?  Additional information on the definitions of other baseline characteristics and potential confounders (lines 218-223) would also be helpful. | We have clearly stated how the socio-economic status quintiles were determined.  Wealth quintiles were calculated from an asset-based index using principal component analysis. The following assets and house characteristics were considered: -  Cupboard, bicycle, radio, mobile phone, motorcycle, cement floor, iron sheets, burnt bricks, and land ownership  We have defined some characteristics, and the rest are self-explanatory for example age and sex of the child. | Page 11  Line 256 to line 260  Page  Line 252 to line 256 |
| Could you also explain how you collected information on “who decided when to initiate breastfeeding”? This is a critical variable in your analysis and should be thoroughly detailed in the Methods section. | We have included this in the methods section. | Page 11  Line 238 to line 240 |
| Results |  |  |
| "Supplementary Table 1" is a key table and should be included in the main manuscript rather than as supplementary material. Several important baseline characteristics and potential confounders are missing from Supplementary Table 1 and should be added: maternal education, occupation, gestational age, household size, and whether the birth was singleton or multiple. | We have included Table 1 in the main manuscript. We have included all these confounders. | Page 15 and page 16 |
| Additionally, including confidence intervals (CIs) or p-values would help interpret the differences between the two groups. | We were instructed to follow the consort statement in writing this manuscript.  The consort statement categorically discourages reporting P-values and any inferential statistics in Table 1.  Below are the some references and explanations to the thinking behind this recommendation  1.M J Knol, R H H Groenwold, D E Grobbee, P-values in baseline tables of randomised controlled trials are inappropriate but still common in high impact journals, European Journal of Preventive Cardiology, Volume 19, Issue 2, 1 April 2012, Pages 231–232, <https://doi.org/10.1177/1741826711421688>  2.Moher D, Hopewell S, Schulz KF, Montori V, Gøtzsche PC, Devereaux PJ, Elbourne D, Egger M, Altman DG. CONSORT 2010 explanation and elaboration: updated guidelines for reporting parallel group randomised trials. Bmj. 2010 Mar 24;340. |  |
| Discussion and conclusion |  |  |
| The authors concluded that the intervention improved exclusive breastfeeding but had no significant impact on the initiation of breastfeeding within the first hour after birth. These conclusions align with the statistical analysis results.  While the discussion is appropriate, it could be strengthened by offering a broader perspective on public health interventions in maternal health.  Specifically, discussing this topic in the context of reproductive rights and maternal autonomy would add valuable depth. | We have edited our discussion and discussed our findings in accordance with your recommendation. | Page 21  Line 409 to line 415 |
| The authors hypothesize (lines 353-354) that the lack of effect on breastfeeding initiation within the first hour may be due to this timing being more influenced by health workers at the facility than by the mother. This is an important point that warrants further exploration: Should health workers receive additional training on the importance of early breastfeeding initiation?  Are there organizational challenges within health facilities that need addressing? This perspective is crucial for future research and public health action and should be developed more fully. | We did not access health workers’ knowledge and practices regarding timely breastfeeding initiation and health facility barriers regarding timely breastfeeding initiation.  We have stated this as a limitation. | Page 24  Line 475 to line 477 |
| Data Availability |  |  |
| The study’s database has been submitted (Excel file) in accordance with PLOS Data Policy, but it requires revisions and additions:  1. The number of observations: n=1898. This includes empty observations and those with only one data point (mum_decide=0). The number of observations should match the number of enrolled pregnant women (n=1877). | This has been addressed. | Need 1877 |
| 2. Additional variables should be included in the database, as it's currently not possible to verify Figure 1 (e.g., “stillbirths,” “child died,” “LTFU”). Additionally, variables such as maternal education and occupation are missing from the Excel file. | This has been addressed. |  |
| A codebook should accompany the database, detailing the variable names and codes. | This has been addressed. |  |
| Please review the coding of variables. For instance, the “marital status” variable needs revision: it is listed as a two-category variable (married/single) in the database but is analyzed as a three-category variable (Supplementary Table 1—married/cohabiting/single). | This has been addressed. |  |
| Minor comments |  |  |
| Line 95: Instead of “less than two-thirds of women in sub-Saharan,” please provide the exact percentage. | Done. | Page 5  Line 96 |
| Line 96: Instead of “two-thirds of mothers in Uganda,” please provide the exact percentage. | Done. | Page 5  Line 97 |
| Line 141: The “map of the study villages in appendix 1” is missing. | We have attached the map as appendix one |  |
| - Figure 1: Please clarify all abbreviations used in the figure (LTFU, EBI, EBF). | We have written all these acronyms in full. | Pge 14  Line 321 |
| - Line 430-431: The phrase “Timely breastfeeding initiation was pro-rich” needs revision for clarity. | We have addressed this. | Page 24  Line 482 and line 484 |

1.	Zeger SL, Liang K-Y, Albert PSJB. Models for longitudinal data: a generalized estimating equation approach. 1988:1049-60.

2.	Moher D, Hopewell S, Schulz KF, Montori V, Gøtzsche PC, Devereaux PJ, et al. CONSORT 2010 explanation and elaboration: updated guidelines for reporting parallel group randomised trials. 2010;340.

3.	Knol M, Groenwold R, Grobbee DJEjopc. P-values in baseline tables of randomised controlled trials are inappropriate but still common in high impact journals. SAGE Publications Sage UK: London, England; 2012. p. 231-2.
