# Supplementary material for: Assessing a bundle of peer counseling, mobile phone messages, and mama kits in promoting timely initiation of and exclusive breastfeeding in Uganda: A cluster randomized controlled study
Source: PLoS One. 2025 Jan 24;20(1):e0317200. doi: 10.1371/journal.pone.0317200 (PMC11761178; doi:10.1371/journal.pone.0317200)
Supplement: Attachment — Submitted filename: Plos__one_response letter_211124_final.docx [file pone.0317200.s008.docx]

Dear Editor,

Trhas Tadesse Berhe, PhD Academic Editor PLOS ONE

Thank you for the opportunity to have our manuscript PONE-D-24-18566

“Assessing a bundle of peer counselling, mobile phone messages, and mama kits in promoting timely initiation of and exclusive breastfeeding in Uganda: a cluster randomized controlled study” reviewed.

We are extremely pleased with the thorough work by the editor and the reviewers, and we want to express our gratitude for your attention to and effort on our manuscript. We have now responded to the reviewer’s submission. Please find our response to the reviewers’ comment below.

| Comment | Response | Page and line number |
| --- | --- | --- |
| Reviewer 5 |  |  |
| As mentioned, 40% of the time it was healthcare worker taking decision for the first hour breastfeeding. I am curious to know, why would a healthcare worker not advice in favor of early initiation. Is it due to inadequate staffing to assist mother for early initiation? or some other reason? I wish to understand the reason behind failure to initiate early breastfeeding. Did you try to explore them? | Unfortunately, we did not explore the reasons behind this phenomenon. For sure, we have inadequate staffing in these health facilities. Each shift has only one midwife on duty, and they often attend to about 10 women in labour. As such, supporting a woman to initiate breastfeeding within the first hour becomes a secondary task that is attended to once all other tasks are done. Also, we cannot rule out a lack of knowledge and poor attitudes towards timely breastfeeding. These are heavy accusations that we did not systematically explore. We have added this is a limitation. | Page 24, line 473-475 |
